# A catalytic chiral gel microfluidic reactor assembled *via* dynamic covalent chemistry†
†Electronic supplementary information (ESI) available: Experimental details, additional characterization and catalytic data. See DOI: 10.1039/c5sc00314h



**DOI:** 10.1039/c5sc00314h

**Published:** 2015-02-18

**Authors:** Haoliang Liu, Juan Feng, Jianyong Zhang, Philip W. Miller, Liuping Chen, Cheng-Yong Su

**Affiliations:** a MOE Laboratory of Bioinorganic and Synthetic Chemistry , MOE Key Laboratory of Polymeric Composite and Functional Materials , Lehn Institute of Functional Materials , School of Chemistry and Chemical Engineering , Sun Yat-Sen University , Guangzhou , 510275 , China . Email: zhjyong@mail.sysu.edu.cn ; Email: cesscy@mail.sysu.edu.cn; b Department of Chemistry , Imperial College London , London , SW7 2AZ , UK . Email: philip.miller@imperial.ac.uk

## Abstract

A novel dynamic covalent gel strategy is reported to immobilize an asymmetric catalyst within the channels of a microfluidic flow reactor. A layer of a catalytically active Mn–salen dynamic covalent imine gel matrix was coated onto a functionalized capillary. Mn–salen active moiety was incorporated into dynamic covalent imine gel matrix *via* the reaction of a chiral Mn–salen dialdehyde unit with a tetraamine linker. The catalytic activity of the capillary reactor has been demonstrated in enantioselective kinetic resolution of secondary alcohols.

## Abstract 2

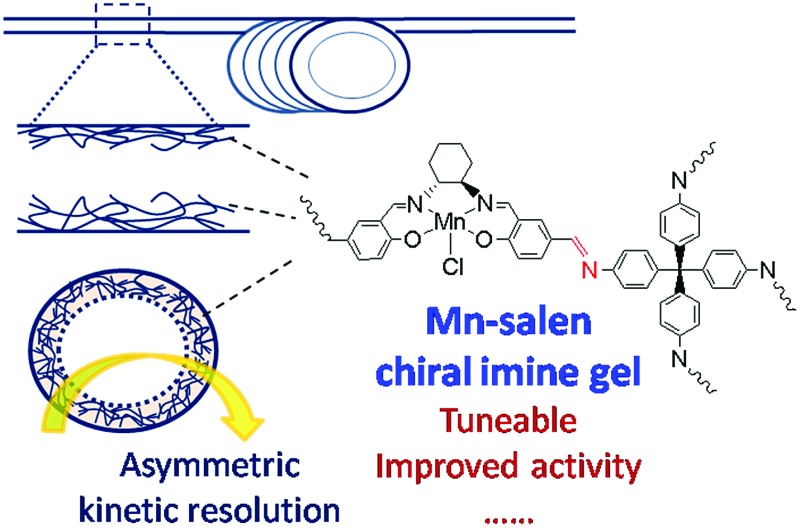
A novel dynamic covalent gel strategy is reported to immobilize an asymmetric catalyst within the channels of a microfluidic flow reactor.

## Introduction

Microfluidic chemical reactors, flow reactors and other small scale continuous process technologies are challenging traditional synthetic chemistry practices.^1^ These flow based reactors are characterised by their small internal volumes (typically ∼100 μL–10 mL), large surface area-to-volume ratios and stable laminar flow profiles. Such flow chemistry systems have key advantages in terms of safety, scalability and improving yields/selectivities compared to traditional batch scale synthetic methods. The small channel diameters (typically ∼10–500 μm) of these microfluidic reactors greatly enhances the available surface area compared to traditional batch reactors which is particularly useful for exploiting reactions or interactions at surfaces. Microfluidic channels are therefore exceptionally useful for supporting reagents, molecular sensors and (chiral) catalysts for flow type processes.^2,3^

Supported catalysts aim to combine the high activities of homogeneous catalysts with the ease of product separation and reusability of heterogeneous catalysts.^4^ As a novel type of supported catalysts, gel catalysts^5–7^ have some advantages as supported catalysis, such as high hierarchical porosity, highly tuneable and unique molecular environment.^8^ A combination of chiral gel catalysts and microfluidic techniques may enhance the catalytic system. For microfluidic techniques the short trans-axial distances and laminar flow pattern typically generate high surface area contact between the liquid phase and supported catalyst to facilitate substance exchange. The active sites at the surface of the channel are exposed to the solution and the flow in the narrow channel can efficiently bring the reactants to the surface and take the products away.^9^ In addition, microfluidic reactors with wall supported catalysts have greater flow stability, lower pressure requirement, and fewer channel blockages^10^ compared to those with packed catalysts,^11^ while no mechanical stirring or agitation would avoid the mechanical degradation of the supported catalysts. Gels may offer the microfluidic devices continuous, hierarchically porous structures with both large flow-through pores and smaller meso- and micropores. In comparison with mesoporous materials supported on capillary wall,^12^ the large pores of gels may enable more effective mass transfer especially for large chiral organic molecules.

Herein we report a novel method for supporting a chiral catalytically active Mn–salen gel onto the surface of microfluidic capillary reactor and demonstrate its potential in enantioselective kinetic resolution of secondary alcohols.^13^ Metal–salen complexes, where the salen backbone is chiral, are well known catalysts for asymmetric reactions.^14,15^ Our gel material relies on the formation of dynamic covalent imine bonds^16,17^ and was designed and self-assembled from a Mn–salen-derived bridging aldehyde and a tetraamine linking unit. It was our aim to coat the inside walls of a capillary with this material, controlling the thickness of the gel deposited, and assess its catalytic activity for enantioselective kinetic resolution reactions.

## Results and discussion

**Fig. 1:**
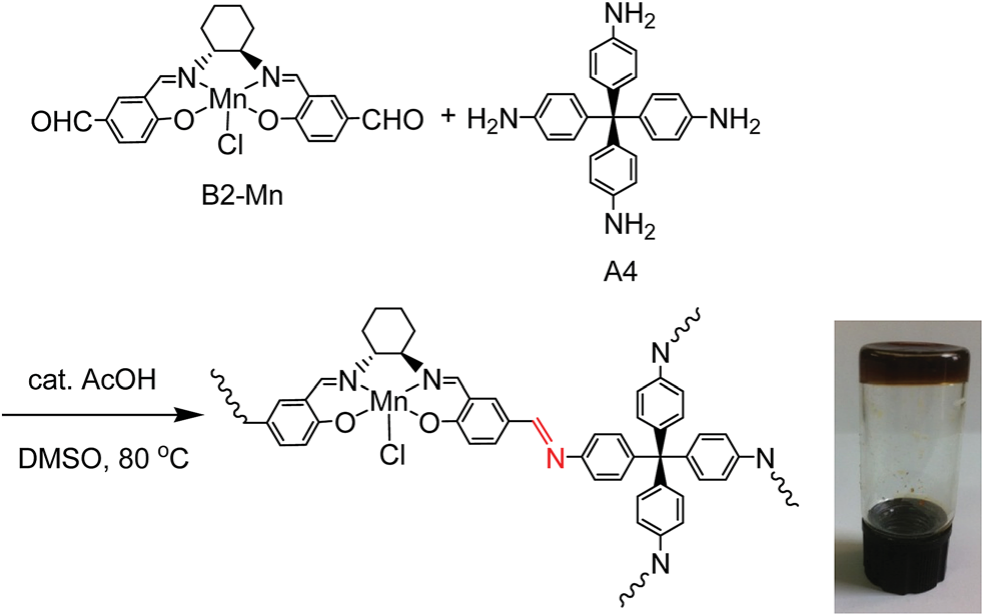
Reaction of B2–Mn and A4 to form chiral Mn–salen imine gel, and photographic image of Mn–salen imine gel (0.02 mol L^–1^ in DMSO).

Mn(iii)–salen ligand, B2–Mn, functionalized with two aldehyde functional groups was synthesized by a Schiff base condensation reaction between (*R*,*R*)-cyclohexanediamine and 4-hydroxy-1,3-benzenedicarboxaldehyde, and then metalated with Mn(OAc)_2_·4H_2_O followed by *in situ* oxidation in air. Reactions of B2–Mn and tetrakis-(4-aminophenyl)-methane (A4) with a molar ratio of 2 : 1 in the presence of a catalytic amount of acetic acid in DMSO at 80 °C afforded a brown Mn–salen imine gel after 1–2 days (Fig. 1). The gel could be obtained when B2–Mn concentration was in the range of 0.02–0.04 mol L^–1^. When the concentration was lower (*e.g.*, 0.01 mol L^–1^), a solution was obtained instead, while higher concentration (*e.g.*, 0.08 mol L^–1^) resulted in precipitation. As a representative the Mn–salen imine gel with B2–Mn concentration 0.02 mol L^–1^ was subjected for further study.

**Fig. 2:**
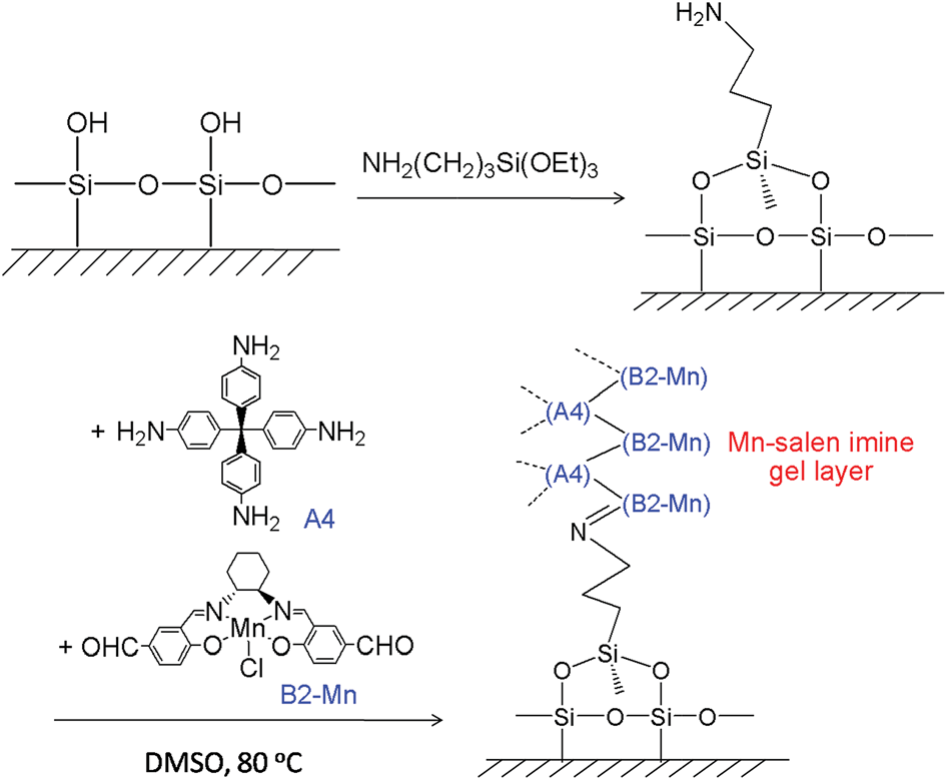
Schematic representation of coating of Mn–salen imine gel on the wall of fused-silica capillary.

**Fig. 3:**
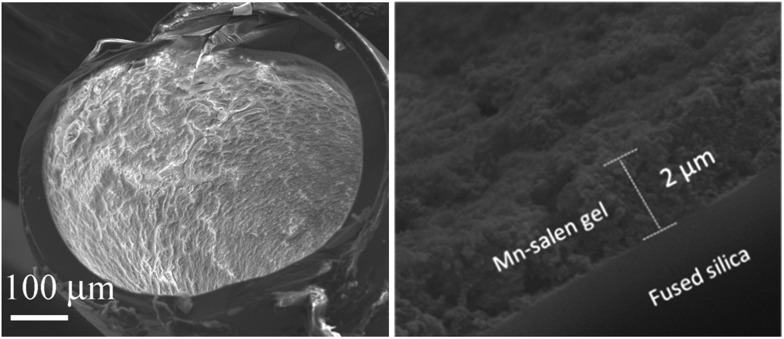
SEM images of a cross-section of a capillary (ID 0.53 mm) coated with Mn–salen imine gel with about 2 μm thickness.

The Mn–salen imine gel was readily introduced into a fused-silica capillary reactor *via in situ* gelation. The preparation method of a gel-coated capillary microreactor is presented in Fig. 2. Firstly the surface of the inner wall of the capillaries was modified with amine functional groups in order to anchor the Mn–salen imine gel *via* imine bonding. The Mn–salen imine gel could be formed inside the capillary after B2–Mn, A4 and AcOH precursor solutions were mixed and heated. To ensure coating of the gel layer on the capillary wall, the precursor solution mixture was allowed to react at room temperature and the excess solution was removed, then the attached solution mixture on the capillary wall was heated at 80 °C to form the Mn–salen imine gel. The thickness of the gel layer was tuneable by repeating the procedure (Fig. S1†). After the procedure was repeated for five times a gel layer with *ca.* 2 μm thickness (evidenced by SEM) was coated on the inside wall of the capillary (Fig. 3).

**Fig. 4:**
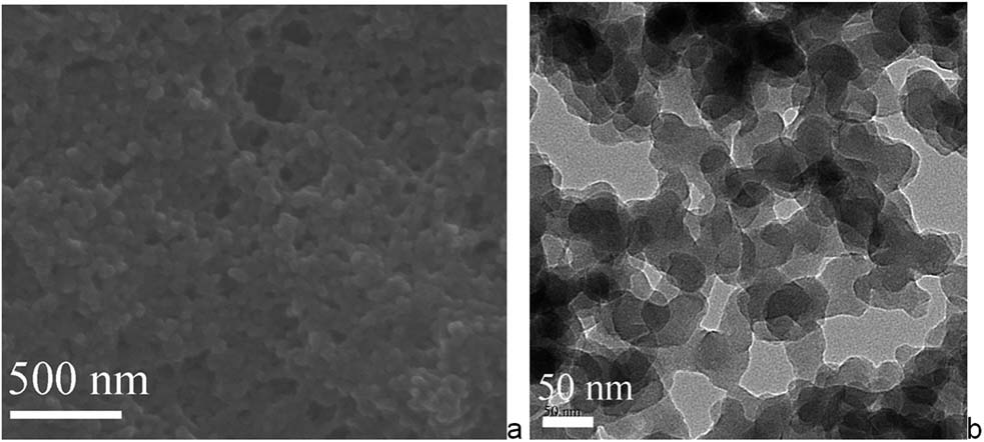
(a) SEM and (b) TEM images of Mn–salen imine gel (0.02 mol L^–1^ in DMSO).

SEM and TEM of the Mn–salen imine gel phase revealed that the tetraamine and dialdehyde precursors, A4 and B2–Mn, polymerize to form primary nanoscale particles with *ca.* 30–50 nanometer size (Fig. 4). The nanoparticles further aggregate *via* imine bonding to form the three-dimensional hierarchically porous gel matrix. The X-ray powder diffraction revealed that the primary nanoparticles are amorphous (Fig. S2†). Thermogravimetric analysis (TGA) showed that the xerogel was stable up to about 300 °C and after 300 °C a significant weight loss was observed (Fig. S3†). FT-IR spectroscopy showed that the aldehyde C=O stretching band at around 1690 cm^–1^ almost vanished (Fig. S4†). The stretching band of newly formed –C=N– imine bond was located at 1611 cm^–1^, overlapping with the salen imine bond. These results confirmed the formation of dynamic covalent imine bonding between the tetraamine and dialdehyde precursors, showing imine bonding is the driving force of gelation.^16^ Elemental composition analysis by energy dispersive X-ray spectroscopy (EDX) revealed the presence of Mn and Cl (Fig. S5†). The presence of trivalent Mn(iii) was confirmed by X-ray photoelectron spectroscopy (XPS) (Fig. S6†). Two signals at 642.5 and 654.4 eV in the Mn 2p_3/2_ and 2p_1/2_ region revealed coordination of Mn(iii) to the ligand. Mn–salen imine gel was dispersed in MeCN and subjected for circular dichroism (CD) investigation (Fig. S7†). Negative CD signal was detected showing the chiral nature of the gel arising from the chiral Mn–salen moieties. The viscoelastic nature of Mn–salen imine gel was confirmed by rheological measurements (Fig. S8†). Storage modulus (*G*′) was higher than loss modulus (*G*′′), indicating presence of a gel phase. To characterize the porosity of the Mn–salen imine gel phase nitrogen physisorption was performed for the corresponding aerogel at 77 K. The aerogel was produced by subcritical CO_2_(l) drying of the wet gel to minimize changing of the original gel structure. The nitrogen isotherm for the aerogel showed type II adsorption branch with a steep rise at *P*/*P*_0_ > 0.9 (Fig. S9a†). The absence of saturation in the adsorption isotherm may arise from the condensation of nitrogen molecules in interparticular space. Brunauer–Emmett–Teller (BET) analysis derived from the adsorption data revealed that the aerogel has moderate specific surface area of 279 m^2^ g^–1^.^18^ The total specific pore volume is 1.622 cm^3^ g^–1^. The aerogel has a very wide mesopore size distribution up to >80 nm according to nonlocal density functional theory analysis (Fig. S9b†).

**Scheme 1:**
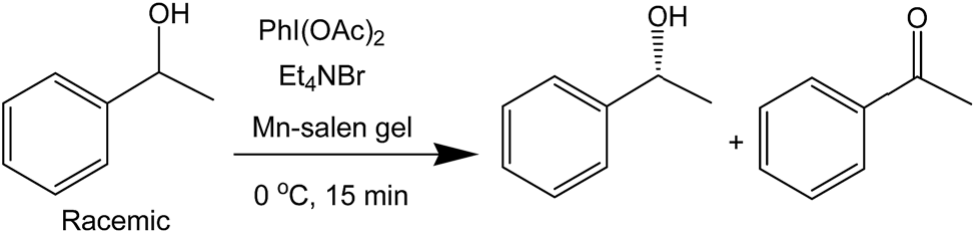
Asymmetric kinetic resolution of (±)-1-phenylethanol catalyzed by Mn–salen imine gel.

**Fig. 5:**
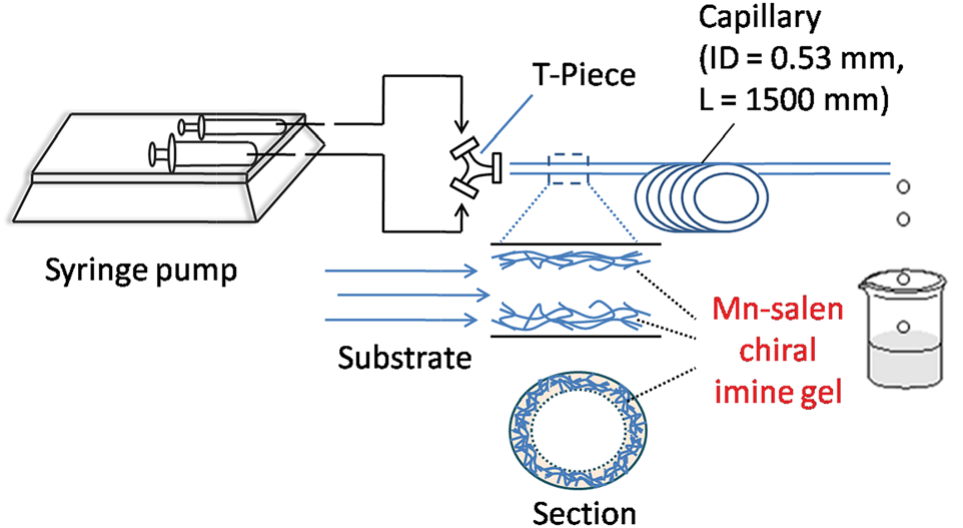
Schematic representation of the microreactor setup using a capillary for asymmetric kinetic resolution of secondary alcohols.

The catalytic activity of the Mn–salen gel microfluidic system was demonstrated in asymmetric kinetic resolution of a series of racemic secondary alcohols. Mn(iii)–salen complexes have been previously studied as catalyst under various homogeneous catalytic conditions^19^ and polymer supported catalytic conditions.^20^ Mn–salen imine gel was used as catalyst with PhI(OAc)_2_ and Et_4_NBr as co-oxidant in CH_2_Cl_2_–H_2_O biphase solvent system. DMSO solvent in the gel catalyst was exchanged to CH_2_Cl_2_ prior to catalysis. 1-Phenylethanol was investigated as a model test substrate (Scheme 1). The reaction system was assembled as shown in Fig. 5. Specifically a DMSO solution of 1-phenylethanol and PhI(OAc)_2_ in CH_2_Cl_2_ and an aqueous solution of Et_4_NBr were introduced using syringes under the control of syringe pumps. The biphase solutions was mixed at the T-piece and then passed the capillary reactor. Capillaries with 2 μm gel layer and various ID (0.53 and 0.25 mm) and length (500–3000 mm) were examined for the kinetic resolution (Table S1†). The best performance was achieved when the reaction mixture was allowed to pass a capillary of 1500 mm long with 0.53 mm in diameter during a period of 15 minutes at 0 °C. Its Mn content was *ca.* 0.027 mmol determined by inductively coupled plasma (ICP) analysis. Under the optimal conditions, (±)-1-phenylethanol was kinetically oxidized in 65% conversion and a product enantiomeric excess (ee) of 91% was observed. Thus a biphase microfluidic system has been successfully demonstrated for enantioselective kinetic resolution.

The asymmetric kinetic resolution is applicable to other racemic secondary alcohols with substitution at *para* position of 1-phenylethanol (Table S2†). For electron withdrawing groups (bromo, chloro and fluoro groups) on the phenyl ring of the substrate higher ee values (70–88%) of the alcohol were achieved (Table S2,† entries 2–4). For 1-(4-methylphenyl)ethanol with electron donating group the ee value decreased to 35% (Table S2,† entry 5). For 4-phenyl-2-butanol with less bulkier groups good ee 91% was achieved (Table S2,† entry 6). The capillary reactor was also effective for 1-indanol, a secondary alcohol with fused-rings, and 62% ee was achieved (Table S2,† entry 7). In contrast due to the presence of two bulkier groups in 1-phenyl-1-propanol low ee 38% was obtained (Table S2,† entry 8). The catalytic activity of the present gel system is generally on parr with the earlier reports on asymmetric kinetic resolution of secondary alcohols with other Mn–salen homogeneous catalysts.^19,20^

The present Mn–salen gel microfluidic system facilitates the separation of the catalyst from the reaction products, allowing simple catalyst recovery and recycling. No Mn was detected in the product solutions as shown by ICP analysis, thus the gel material is stable under these flow conditions and does not leach Mn–salen. The capillary reactor could be readily reused for the next run after flushing with CH_2_Cl_2_ (3 mL). The system is robust with no significant loss of reactivity or enantioselectivity over at least eight runs (Table S3†).

The capillary reactor system was compared to a number of control experiments. First an analogous reaction was performed in a flask in order to compare the behaviour of the gel catalyst under batch conditions. The reactions were tested over a range of different catalyst loading (Fig. S10†). Optimal catalyst performance for these reactions was found to be in the range 1.5–4.0 mol% over *ca.* 120 min. Under these optimal conditions conversions were in the range 63–68% and ee 91–93% of the formed products. The reaction could not be further improved by either increasing catalyst loadings or extending the reactions times. The Mn–salen imine gel catalyst can be readily recovered by centrifugation and reused up to five times without significant losses in conversion or ee. Conversions ranged 63%–66% and ee 93–90% over a 120 min reaction time (Fig. S11†). In comparison to the batch catalytic process, the capillary reactor achieves similar conversions and ee in much shorter reaction times.

The catalytic performance of the capillary reactor was also compared with that of B2–Mn precursor. Under homogeneous conditions the kinetic resolution of 1-phenylethanol was achieved by B2–Mn catalyst with 60% conversion 83% ee in 120 minutes (Fig. S12†). We were initially surprised at the difference in activity of the homogeneous and heterogeneous reactions compared to the capillary method. We hypothesise that this may be attributed to reagents interacting with a much larger proportion of the gel material under capillary reactor conditions (due to the much larger surface area exposed in the reactor) than that of in the batch methods. Additionally the gel phase traps the chiral active centre in a rigid matrix which improves mass transfer.

This was further supported by using the corresponding xerogel catalyst inside capillary. The capillary reactor coated with Mn–salen imine xerogel was prepared by drying the reactor in vacuum. The xerogel has significantly decreased (meso-/macro-) porosity (*S*_BET_ = 162 m^2^ g^–1^, total specific pore volume 0.318 cm^3^ g^–1^, Fig. S9†) compared to the wet gel. For the reaction of 1-phenylethanol 58% substrate conversion and 85% product ee were achieved. Comparison of the results also showed that the wet gel capillary reactor exhibited higher product enantioselectivity.

## Conclusions

In summary, we have prepared a novel capillary microreactor coated with a chiral Mn–salen imine gel. The dynamic covalent Mn–salen imine gel was synthesized *via* the reaction of a Mn–salen dialdehyde with a tetraamine and attached *via* an amine to the capillary surface. It represents a catalogue of dynamic covalent gels that are expected to be highly tuneable given the rich diversity of dynamic covalent chemistry. The Mn–salen gel, under batch reaction conditions and the capillary-gel reactor have proven effective for the enantioselective kinetic resolution of secondary alcohols, giving good conversions and ee. Under the gel-capillary flow reaction conditions optimal conversions were achieved in much shorter reaction times (15 min) compared to the batch process (120 min). In addition, the capillary reactor could be reused at least eight times without loss of activity or enantioselectivity. We believe this type of reactor may have great potential for high-throughput screening of reactions. Using this gel capillary-immobilization strategy it should be possible to develop a wide range of supported catalysts directly and in a controlled manner, on the surfaces of microchannels and thus investigate a much broader range of catalytic reactions. We are currently investigating further applications of these gels and their wider catalytic potential.
