# Supplementary material for: A catalytic chiral gel microfluidic reactor assembled via dynamic covalent chemistry
Source: Chem Sci. 2015 Feb 18;6(4):2292–6. doi: 10.1039/c5sc00314h (PMC5488196; doi:10.1039/c5sc00314h)
Supplement: Supplementary file 1 [file SC-006-C5SC00314H-s001.pdf]

Electronic Supplementary Material (ESI) for Chemical Science.
This journal is © The Royal Society of Chemistry 2015

Electronic Supplementary Information:

A catalytic chiral gel microfluidic reactor assembled via dynamic covalent chemistry

Haoliang Liu, [a] Juan Feng, [a] Jianyong Zhang,*[a] Philip W. Miller,*[b] Liuping Chen[a] and Cheng-Yong Su*[a]

[a] *MOE Laboratory of Bioinorganic and Synthetic Chemistry, MOE Key Laboratory of Polymeric Composite and Functional Materials, Lehn Institute of Functional Materials, School of Chemistry and Chemical Engineering, Sun Yat-Sen University, Guangzhou, 510275, China. E-mail: zhjyong@mail.sysu.edu.cn; cesscy@mail.sysu.edu.cn*

[b] *Department of Chemistry, Imperial College London, London, SW7 2AZ, UK. E-mail: philip.miller@imperial.ac.uk*

Experimental

Starting materials and solvents were obtained from commercial sources and used without further purification unless otherwise stated. 5-chloromethyl-2-hydroxybenzaldehyde[1] and 4-hydroxy-1,3- benzenedicarboxaldehyde[2] were synthesized according to the published methods. Scanning electron microscopy (SEM) and energy dispersive X-ray spectroscopy (EDX) were taken using a Quanta 400F scanning electron microscope with an Inca energy dispersive X-ray spectrometer. Before measurement, the gel samples were dispersed in ethanol, put on aluminum foil, and sputter coated with gold. Transmission electron micrographs were conducted on a FEI Tecnai G2 Spirit 120 kV transmission electron microscope. The samples for TEM observations were prepared by dispersing the gels in ethanol by sonication and then immersing a carbon-coated copper grid. X-ray powder diffraction data were collected on a Bruker D8 ADVANCE diffractometer at 40 kV and 40 mA with a Cu-target tube and a graphite monochromator (λ = 1.5418 Å). Thermo analyses were performed under N_2 atmosphere at a heating rate of 10 ^{o}C min^{-1} with a NETZSCH TG 209 F3 or SHIMADZU TGA-50 system. N_2 adsorption measurements were performed using a Quantachrome Autosorb-iQ_2 analyzer. Before sorption measurements, the aerogel sample was degassed at 100 ^{o}C for 12 h under high vacuum. NMR spectra were obtained on a Bruker Avance III 400MHz or VARIAN Mercury-Plus 300 spectrometer. MS experiments were performed on a Bluker ESI-Q-TOF maxis 4G high-resolution mass-spectrometer. The CD spectrum was recorded on a Jasco J-810 Circular Dichroism Spectrometer. Rheological measurement of the DMSO gel was performed on an ARES/RFS rheometer (TA-WATEYS Instruments). The frequency sweep was obtained from 0.01 rad s^{-1} to 100 rad s^{-1} at a constant strain of 1.0%. HPLC experiments were performed on Agilent 1200 equipped with a Chiralcel OD-H column, 4.6 mm (ID) × 250 mm (L).

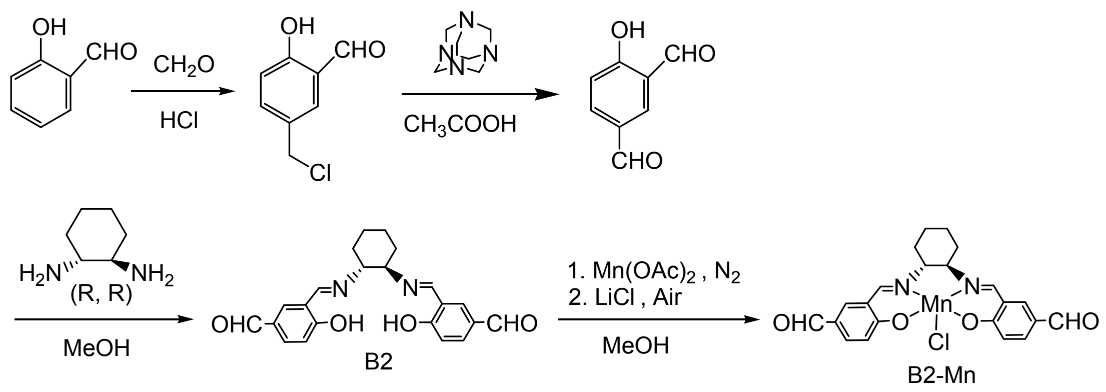

Scheme S1. Synthetic route of B2-Mn.

Synthesis of B2. To a solution of 4-hydroxy-1,3-benzenedicarboxaldehyde (0.795 g, 5.3 mmol, excess) in MeOH (25 mL), (+/-)-trans-1,2-diaminocyclohexane (0.228 g, 2.0 mmol) in MeOH (25 mL) was added dropwise at 50 °C. The resulting solution was stirred for 40 min at 50 °C. After the reaction mixture was cooled down to room temperature, the solvent was evaporated under reduced pressure. The resulting yellow oil was dissolved in MeOH (15 mL) followed by slow evaporation, yielding yellow solid product after 2 days. The yellow solid was filtered, washed with EtOAc, EtOH and dried in vacuum (0.36 g, 38%). M.p. > 220 °C (dec.). Anal. found (calcd.) for $C_{22}H_{22}N_2O_4$: C 69.40 (69.83), H 6.025 (5.86), N 7.25 (7.40)%. ^{1}H NMR (300 MHz, DMSO-d_6): δ 9.69 (s, 2H), 8.65 (s, 2H), 7.92 (d, J = 1.9 Hz, 2H), 7.73 (dd, J = 8.7, 2.1 Hz, 2H), 6.83 (d, J = 8.7 Hz, 2H), 3.62-3.65 (m, 2H), 1.22-1.99 (m, 8H). ^{1}H NMR (400 MHz, $CDCl_3$) δ 14.18 (s, 2H), 9.82 (s, 2H), 8.37 (s, 2H), 7.82 (dd, J = 8.6, 2.1 Hz, 2H), 7.74 (d, J = 2.1 Hz, 2H), 7.01 (d, J = 8.6 Hz, 2H), 3.50–3.39 (m, 2H), 2.05–2.00 (m, 2H), 2.00–1.93 (m, 2H).1.87–1.70 (m, 2H), 1.57–1.48 (m, 2H). ^{13}C NMR (101 MHz, C-H decoupled, $CDCl_3$) δ 189.94 (s), 167.31 (s), 164.24 (s), 134.43 (s), 133.87 (s), 128.00 (s), 118.35 (s), 118.05 (s), 72.05 (s), 32.84 (s), 24.02 (s). TOF-MS (ESI+): *m*/*z* found (calcd.) for $[M+Na]^+$ = 401.1478 (401.1472). FT-IR (cm^{-1}, KBr): 1686 ν(C=O), 1632 ν(C=N).

Synthesis of B2-Mn. To a solution of above synthesized B2 (187 mg, 0.5 mmol) in 20 mL anhydrous methanol, solid $Mn(OAc)_2{\cdot}4H_2O$ (123 mg, 0.5 mmol) was added under N_2 atm. and the resulting solution was refluxed for about 8 h. Then the reaction

mixture was cooled to RT, followed by the addition of LiCl (144 mg, 4.0 mmol) and the stirring was continued for another 5 h under air for the aerial oxidation of Mn(II) to Mn(III). Subsequently, the solvent was evaporated and the residue was extracted with CH_2Cl_2. The organic layer was washed three times with water, twice with brine and dried over anhydrous Na_2SO_4. Finally the solvent was evaporated under reduce pressure to get the Mn(III)-salen complex as brown solid (170 mg, 68%). M.p. > 290 ^{o}C (dec.). Anal. found (calcd.) for $C_{22}H_{20}N_2O_4MnCl{\cdot}3H_2O$: C 50.71 (50.73), H 4.85 (5.03), N 5.10 (5.38)%; TOF-MS (ESI+): *m/z* found (calcd.) for $[M\text{-}Cl]^+$ = 431.0795 (431.0798). FT-IR (cm^{-1}, KBr): 1690 ν(C=O), 1605 ν(C=N).

Preparation of Mn-salen imine gel. To a solution of B2-Mn (9.3 mg, 0.02 mmol) in DMSO (0.5 mL), a solution of tetrakis-(4-aminophenyl)methane (3.8 mg, 0.01 mmol) in DMSO (0.5 mL) was added dropwise whilst stirring to form a solution. To the solution mixture, HAc (30 μL, 3 mol L^{-1} in DMSO) was added. The resulting brown solution was heated in a closed vial at 80 ^{o}C to obtain an opaque brown gel after 2 days. The above resulting gel was further aged for 5 days. The gel was subsequently washed with DMSO at room temperature. DMSO was replaced every day by fresh solvent and solvent exchange was finished after 3 days. Then the gel was washed with anhydrous EtOH for 3 days in similar way.

1) The corresponding xerogel was obtained by drying the gel in vacuum for 12 h (10.6 mg, 85%). Anal. Found (Calcd.) for $C_{69}H_{56}N_8O_4Mn_2Cl_2$: Mn 7.73 (8.85)%.

2) The corresponding aerogel was obtained by extracting the solvent in the exchanged gels with subcritical CO_2(l) in a 0.75 L high pressure stainless-steel Soxhlet extractor (7.4 mg, 58%). Anal. Found (Calcd.) for $C_{69}H_{56}N_8O_4Mn_2Cl_2$: Mn 8.46 (8.85)%. FT-IR (cm^{-1}): 1611 ν(C=N).

Gel attachment in capillaries. Prior to the coating of Mn-salen imine gel, the inner surface of a silica capillary column (L = 5.0 m, ID = 0.53 mm) was modified with amino groups. The capillary was first rinsed with acetone for 30 min. Then the capillary was rinsed with a mixture of 30% H_2O_2 and 30% ammonia (v:v = 1:1) for 4

h. After that the capillary was rinsed with distilled water, ethanol, and Et_2O for 10 min, respectively, and dried in a stream of argon for 4 h. Subsequently the capillary was filled with a solution of 3-aminopropyl-siloxyethane in dry toluene (1%, v:v) and kept for 1 h. Afterwards this solution was removed and the capillary was rinsed with dry toluene and Et_2O for 10 min, respectively, and dried in a stream of argon for 4 h.

The above pre-treated capillary was filled with the solution mixture of a solution of B2-Mn (9.3 mg, 0.02 mmol) in DMSO (0.5 mL), a solution of tetrakis-(4-aminophenyl)methane (3.8 mg, 0.01 mmol) in DMSO (0.5 mL), and HAc (30 μL, 3 mol L^{-1} in DMSO). The capillary was sealed and kept for 4 h at room temperature. Then the solution was removed by flushing the capillary with argon and the capillary was flushed with argon for 30 minutes. The capillary was sealed and heated for 4 h at 80 ^{o}C for 48 h. The capillary was rinsed with DMSO (1 mL × 3). The procedures were repeated for five times to obtain the capillary for catalytic study (the coating thickness is ca. 2 μm according to SEM). Prior to catalysis, DMSO solvent in the gel was exchanged to CH_2Cl_2.

Asymmetric kinetic resolution of racemic secondary alcohols in capillary reactor. In a typical process, solution A was prepared by dissolving (±)-1-phenylethanol (61.0 mg, 0.50 mmol) and $PhI(OAc)_2$ (112.7 mg, 0.35 mmol) in CH_2Cl_2 (1 mL). Solution A and a solution of Et_4NBr (4.2 mg, 0.02 mmol) in water (2 mL) was injected into two separate FEP tubes before T-piece, respectively. Then the two solutions were pushed by pure CH_2Cl_2 and water, respectively to mix and react in the gel-coated capillary (ID = 0.53 mm, L = 1500 mm) at 0 ^{o}C. The flow rate was kept to be 18 μL min^{-1} and the reaction time in the gel-coated capillary was 15 min. The reaction mixture was collected for a period of ca. 1 min and evaporated to dryness. Then hexane (5 mL) was added to the reaction mixture, and the organic layer was subjected to ^{1}H NMR and HPLC analysis to determine the conversion of (±)-1-phenylethanol and the enantiomeric excess (ee) of the product, respectively. Prior to HPLC analysis, the resulting ketone and enantio-enriched secondary alcohol were separated by silica gel

flash chromatography. For subsequent use, the capillary reactor was rinsed with CH_2Cl_2 (1 mL) for three times.

Asymmetric kinetic resolution of racemic secondary alcohols under batch conditions. In a typical process, to a 10 mL glass reactor (±)-1-phenylethanol (0.50 mmol), catalyst (0.01 mmol, 2 mol%), CH_2Cl_2 (1 mL) and H_2O (2 mL) were added and the resulting mixture was stirred for 5 min. To the mixture, Et_4NBr (4 mol%) was added at room temperature. The resulting reaction mixture was next cooled to 0 ^oC and $PhI(OAc)_2$ (0.7 equiv.) was added slowly in small fractions over 30 min and the reaction mixture was stirred for 6 h at 0 ^oC. After the completion of the reaction, substrate and product were separated by the addition of hexane (5 mL) to the reaction mixture. The organic layer was subjected to ^1H NMR and HPLC analysis to determine the conversion of (±)-1-phenylethanol and the enantiomeric excess (ee) of the product, respectively. Prior to HPLC analysis, the resulting ketone and enantio-enriched secondary alcohol were separated by silica gel flash chromatography. For recovering of the gel catalyst, after addition of hexane to the reaction mixture the gel was separated by centrifugation and washed by ethanol and hexane in sequence.

References

1 S. Sonar, K. Ambrose, A. D. Hendsbee, J. D. Masuda and R. D. Singer, *Can. J. Chem.*, 2012, **90**, 60-70.

2 F. M. Mei, L. J. Chen and G. X. Li, *Appl. Organometallic Chem.*, 2010, **24**, 86-91.

Table S1. Asymmetric kinetic resolution of (±)-1-phenylethanol in the gel capillary reactor with various ID and length capillaries (substrates have 15 min residence time; gel layer ca. 2 μm).

entry	ID/mm	Length/mm	Conversion/%[a]	ee/%[b]
1	0.25	1500	55	86
2	0.25	3000	58	91
3	0.53	500	61	72
4	0.53	1000	57	89
5	0.53	1500	65	91

[a] Determined by ^{1}H NMR. [b] Determined by HPLC with Daicel Chiralcel OD-H column.

Table S2. Asymmetric kinetic resolution of various racemic secondary alcohols in the gel capillary reactor (substrates have 15 min residence time in the capillary, ID 0.53 mm × 1500 mm).

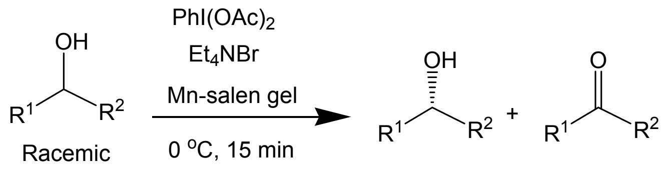

entry	substrate	conversion/%[a]	ee/%[b]
1	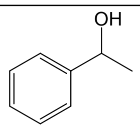	65	91
2	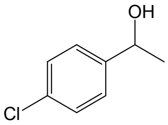	48	70
3	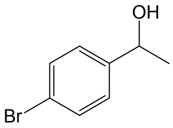	58	88
4	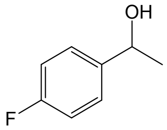	65	82
5	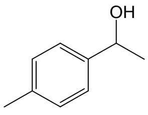	51	35
6	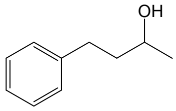	59	91
7	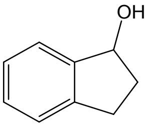	51	62
8	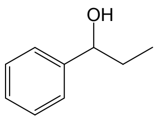	46	38

[a] Determined by ^1H NMR. [b] Determined by HPLC with Daicel Chiralcel OD-H column.

Table S3. Catalytic activity of eight runs of asymmetric kinetic resolution of (±)-1-phenylethanol in the gel capillary reactor (substrates have 15 min residence time in the capillary, ID 0.53 mm × 1500 mm).

run	1	2	3	4	5	6	7	8
conversion/%[a]	65	65	64	66	63	63	64	60
ee/%[b]	91	90	92	91	90	89	90	90

[a] Determined by ^1H NMR. [b] Determined by HPLC with Daicel Chiralcel OD-H column.

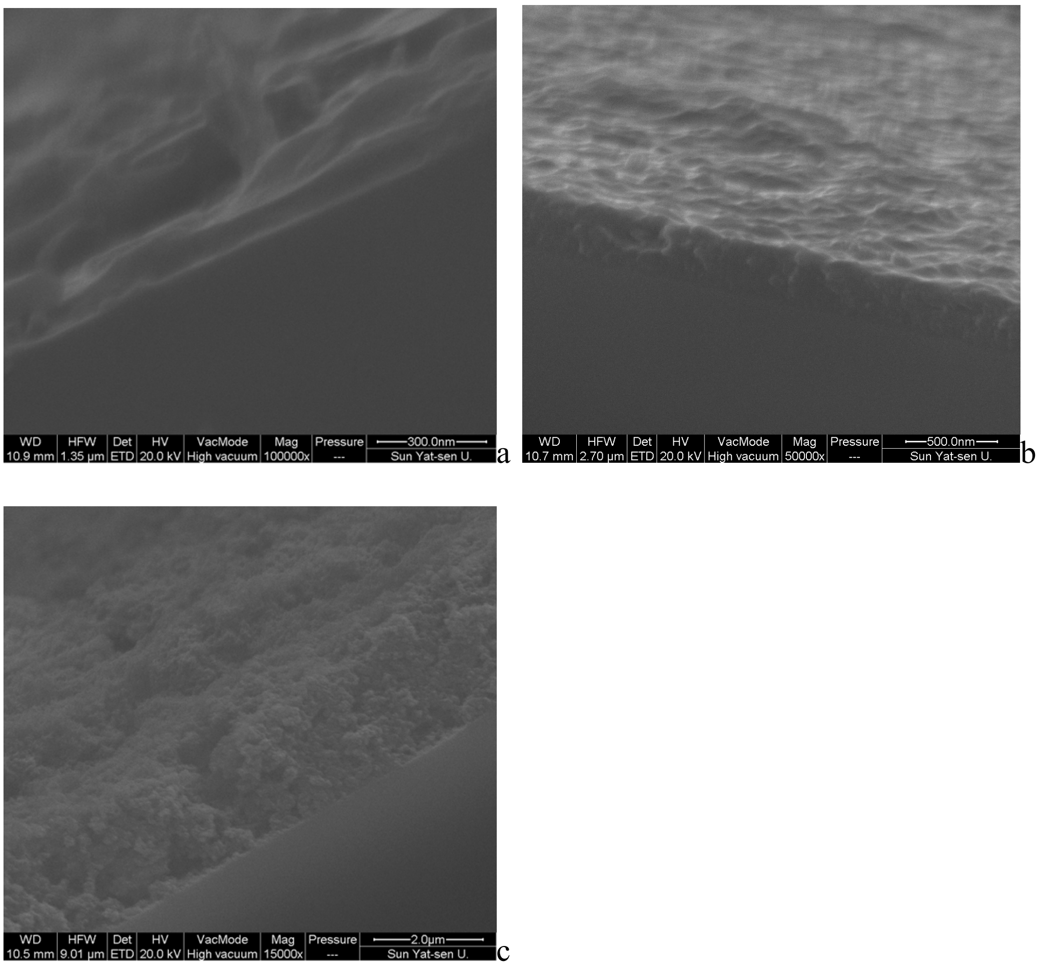

Figure S1. SEM images of the cross-sections of capillaries (ID 0.53 mm) coated with Mn-salen imine gel with increasing thickness by repeating the coating procedure for a) once, b) three times and c) five times.

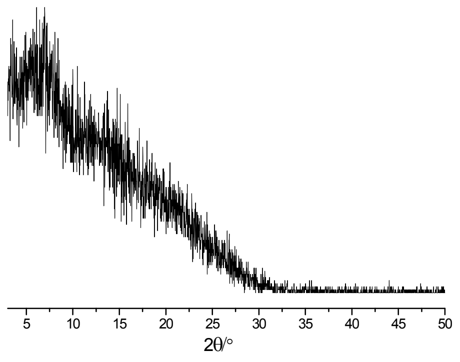

Figure S2. XRPD pattern of Mn-salen imine aerogel.

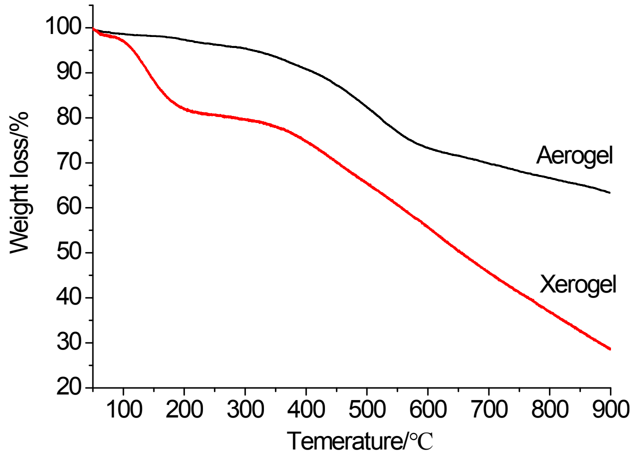

Figure S3. TG curves of Mn-salen imine aerogel and xerogel.

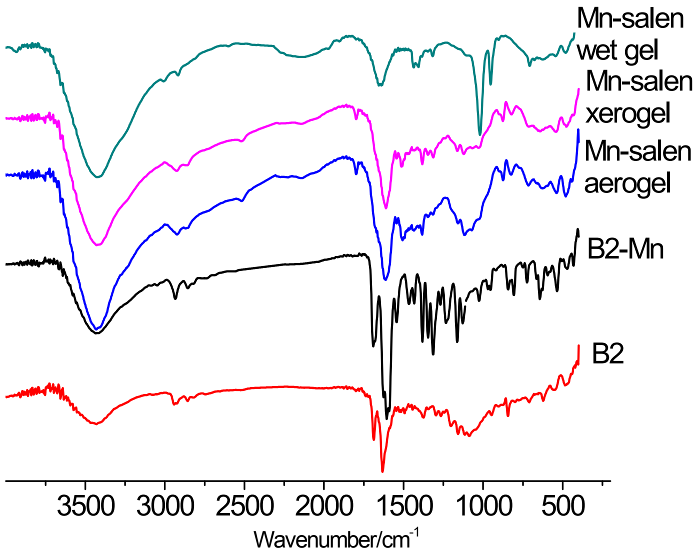

Figure S4. FT-IR spectra of B2, B2-Mn and Mn-salen imine gel.

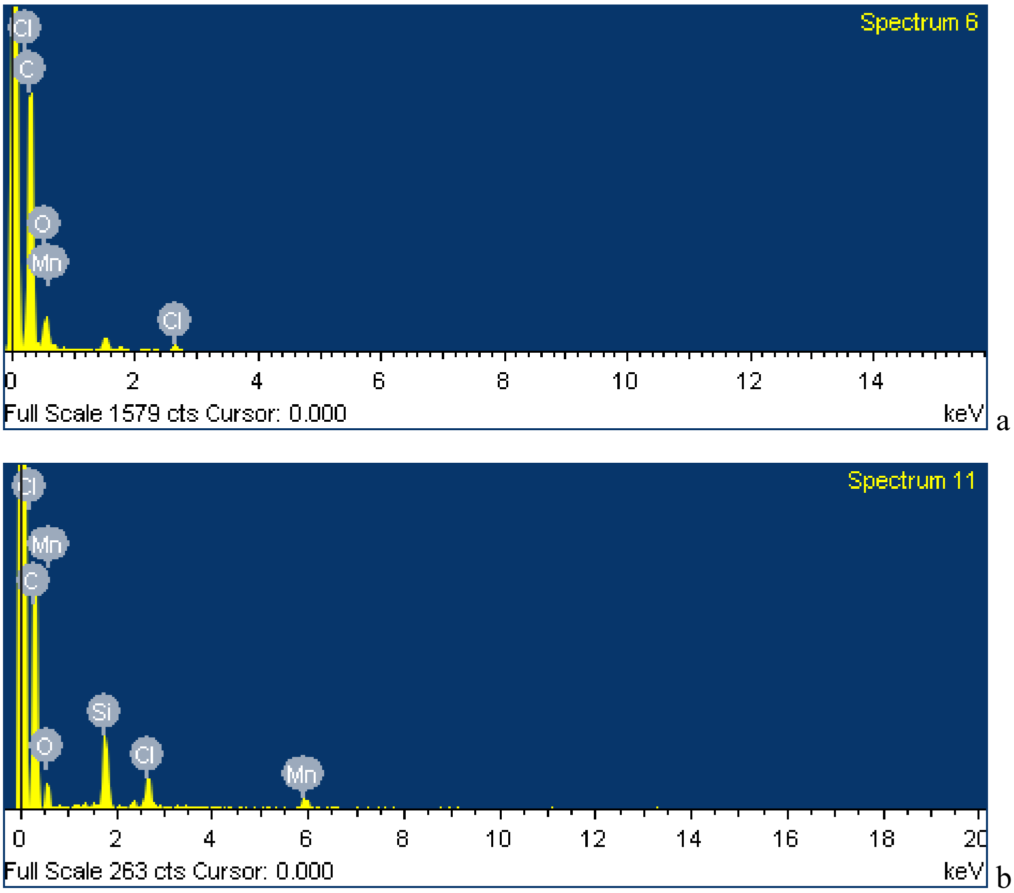

Figure S5. EDX spectra (no Au coating) of a) Mn-salen imine aerogel showing atomic composition of C 77.83, O 21.93, Mn 0.03 and Cl 0.22%, b) the coating Mn-salen imine gel layer on the capillary wall showing atomic composition of C 84.44, O 12.20, Si 1.90, Mn 0.62 and Cl 0.84%, indicating attachment of Mn-salen imine gel.

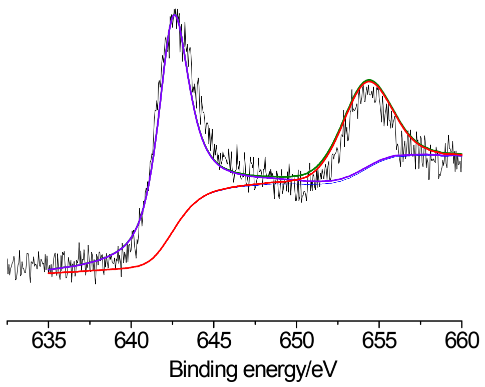

Figure S6. XPS Mn 2p spectrum and the deconvoluted spectra of Mn-salen imine aerogel.

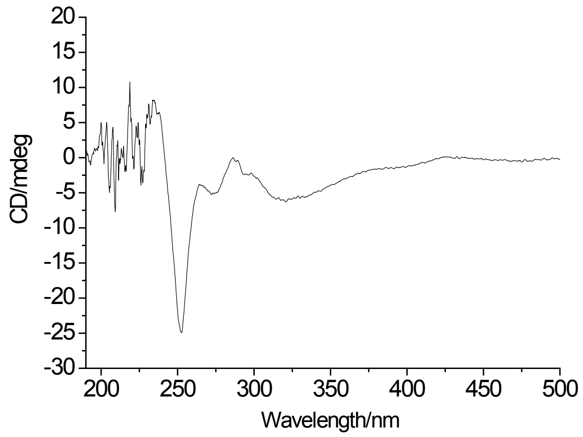

Figure S7. CD spectra of Mn-salen imine gel dispersed in MeCN at room temperature.

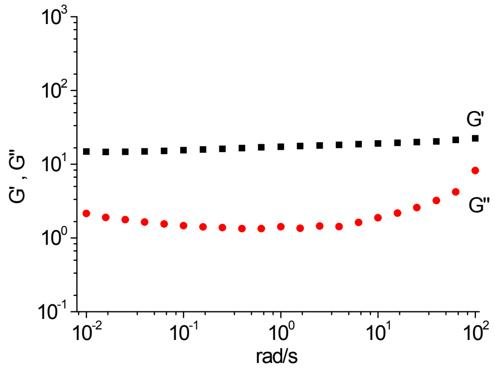

Figure S8. Variation of storage modulus (*G*') and loss modulus (*G*'') with frequency for Mn-salen imine gel.

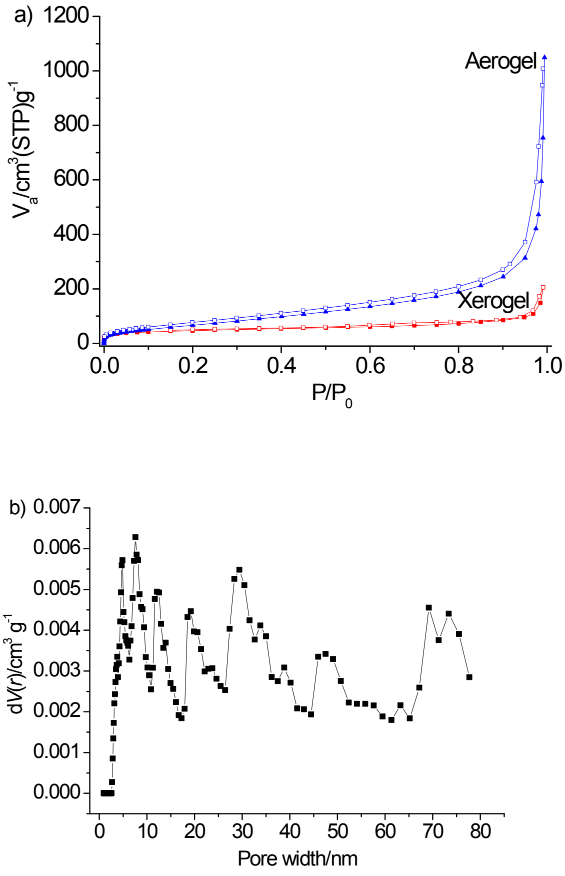

Figure S9. a) N_2 adsorption and desorption isotherms of Mn-Salen imine aerogel and xerogel at 77 K and b) pore size distributions of Mn-Salen imine aerogel calculated by application of nonlocal density functional theory (NL-DFT) (with cylindr pore silica model).

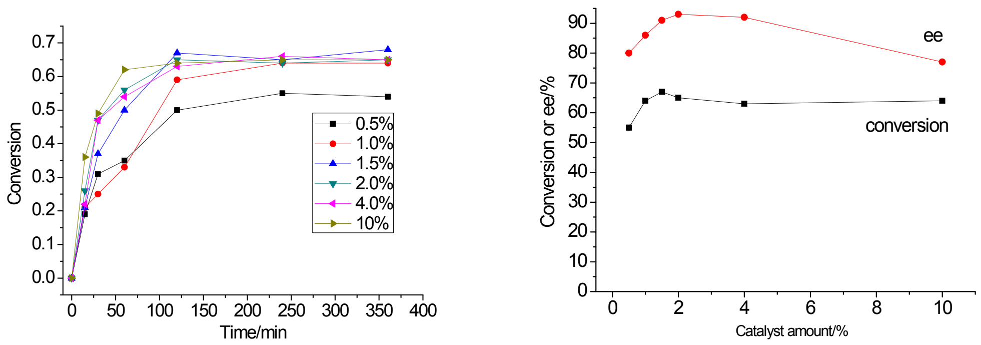

Figure S10. Asymmetric kinetic resolution of (±)-1-phenylethanol catalysed by Mn-salen imine gel under batch conditions with various catalyst amount (When the catalyst amount was 10%, the reaction mixture was slurry-like), a) conversion vs time curves, and b) conversion and ee after 120 minutes.

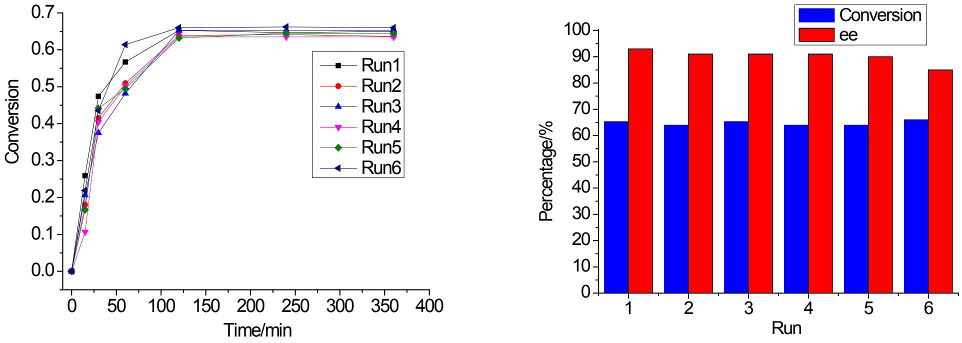

Figure S11. Effect of the catalyst (Mn-salen imine gel) recycling (six runs) on asymmetric kinetic resolution of (±)-1-phenylethanol under batch conditions, a) conversion vs time curves, and b) conversion and ee after 120 minutes.

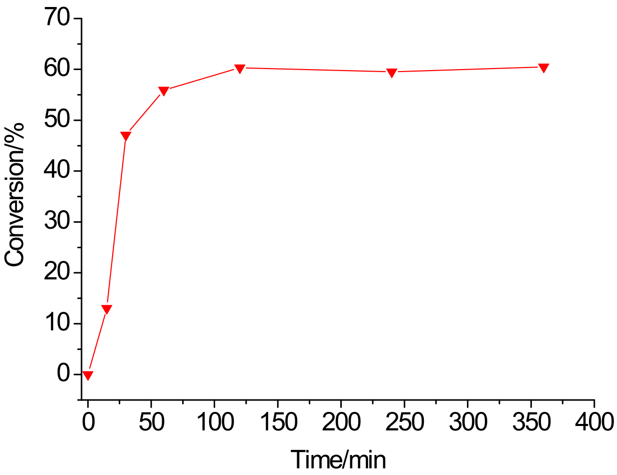

Figure S12. Asymmetric kinetic resolution of (±)-1-phenylethanol catalysed by B2-Mn under homogeneous conditions.

NMR and MS spectra of B2 and B2-Mn.

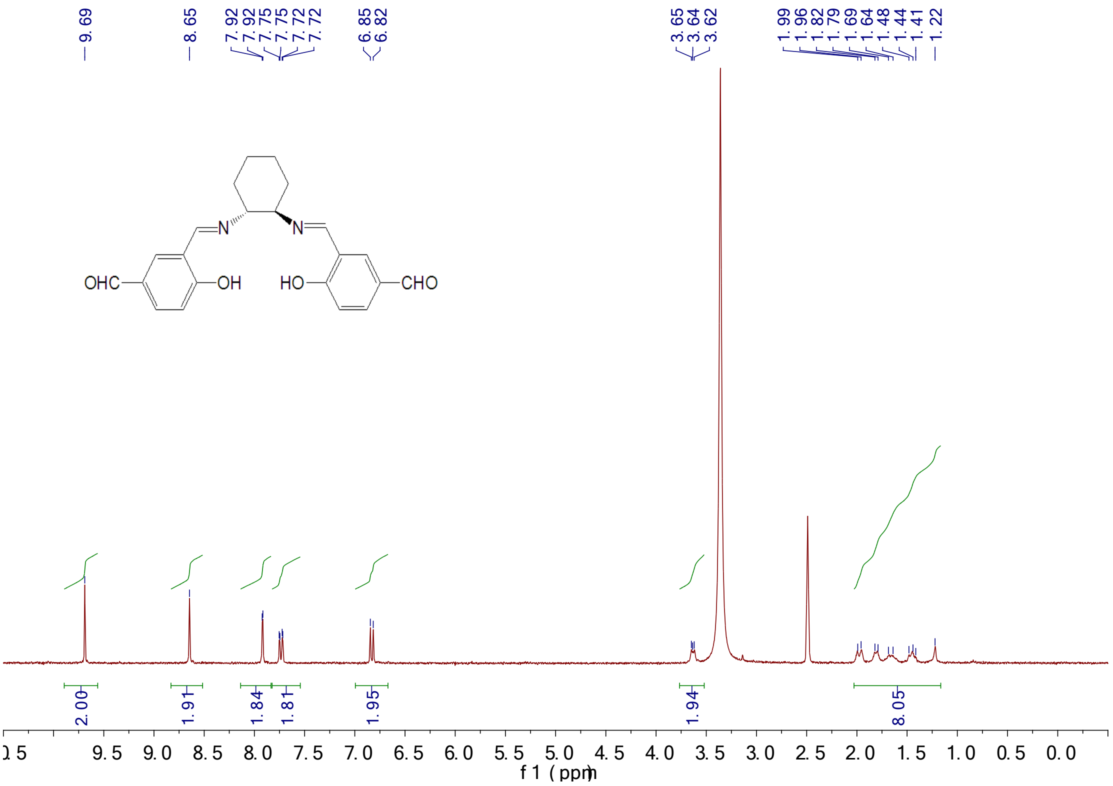

Figure S13. The ^{1}H NMR spectrum of B2 (300 MHz, DMSO-d_6).

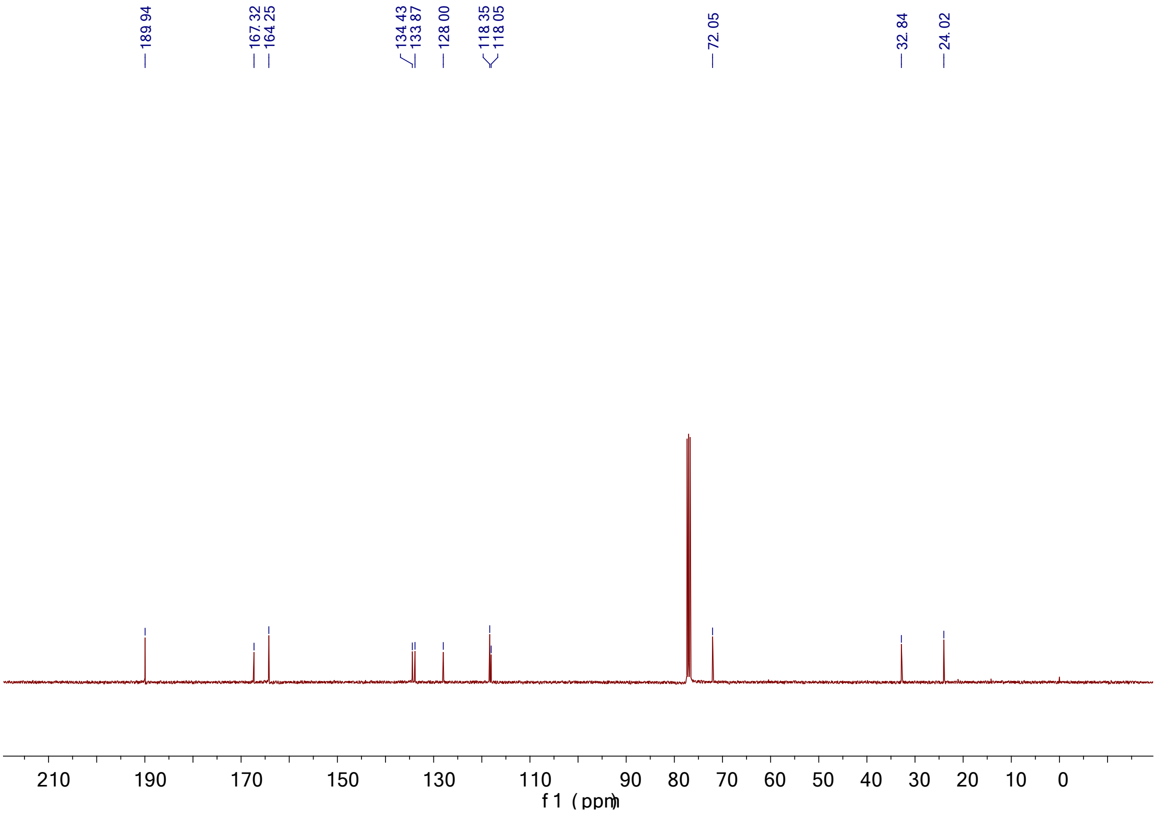

Figure S14. The ^{13}C NMR spectrum of B2 (101 MHz, $CDCl_3$).

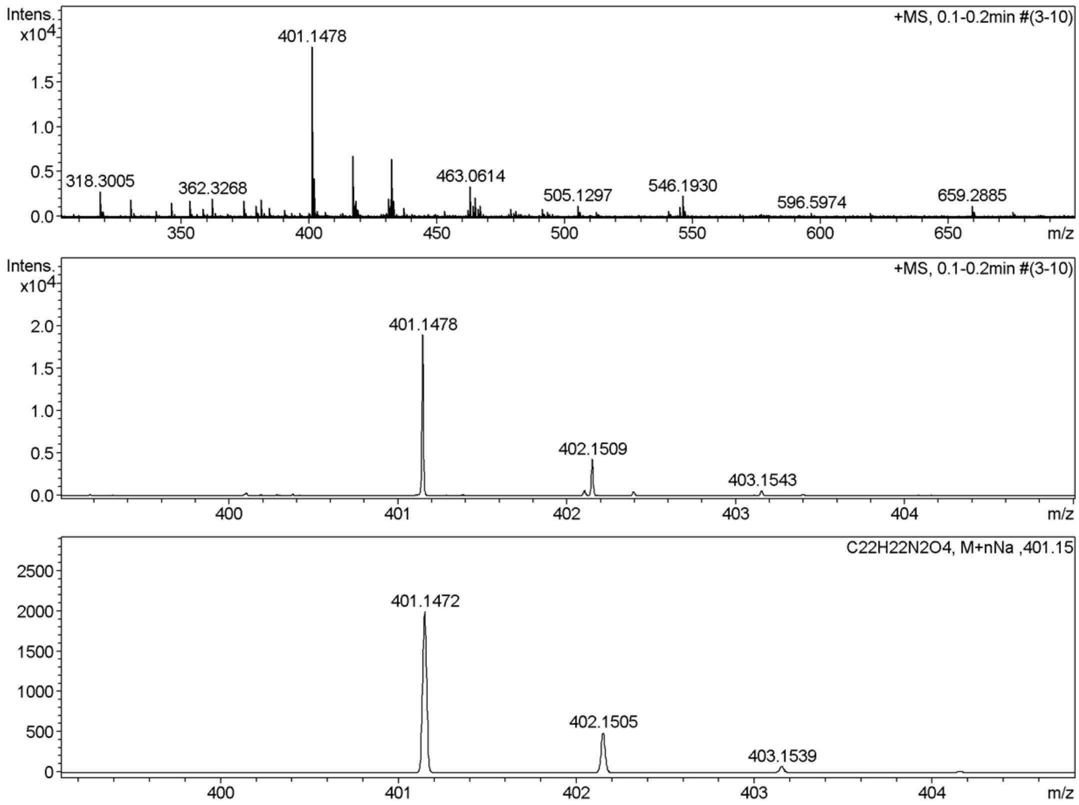

Figure S15. The ESI-MS spectrum of B2 in MeOH, and isotopic distribution and simulation of $[M + Na]^+$.

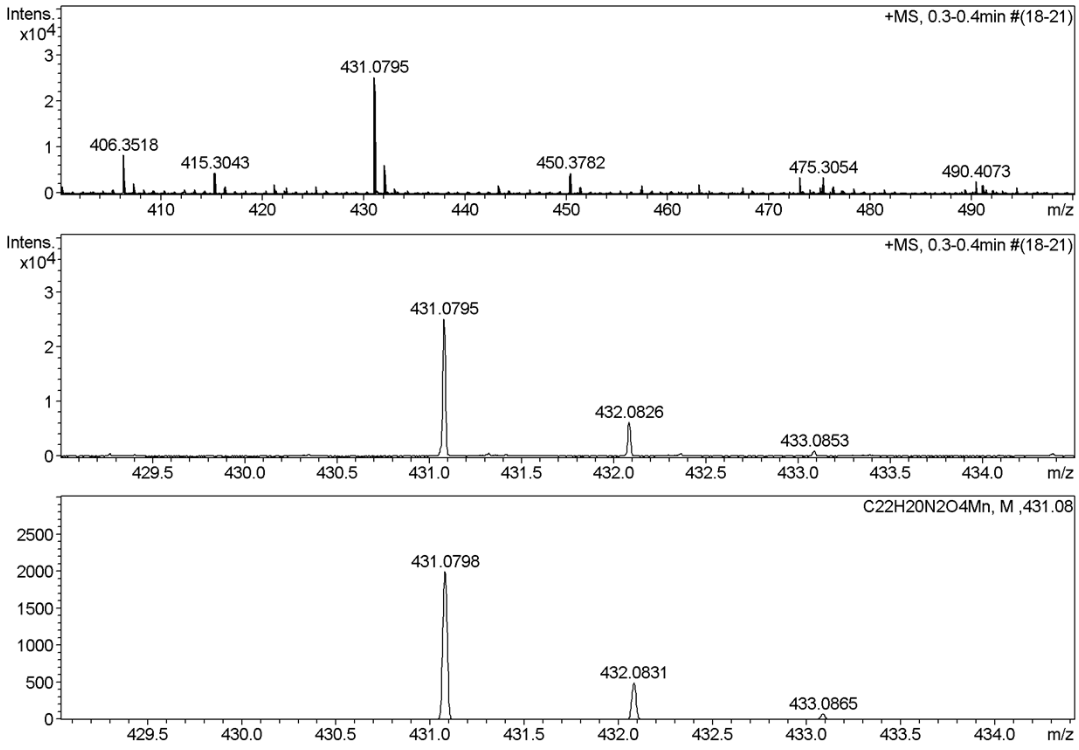

Figure S16. The ESI-MS spectrum of B2-Mn in MeOH, and isotopic distribution and simulation of $[M - Cl]^+$.

HPLC profiles of racemic secondary alcohols resolved by the gel capillary reactor.

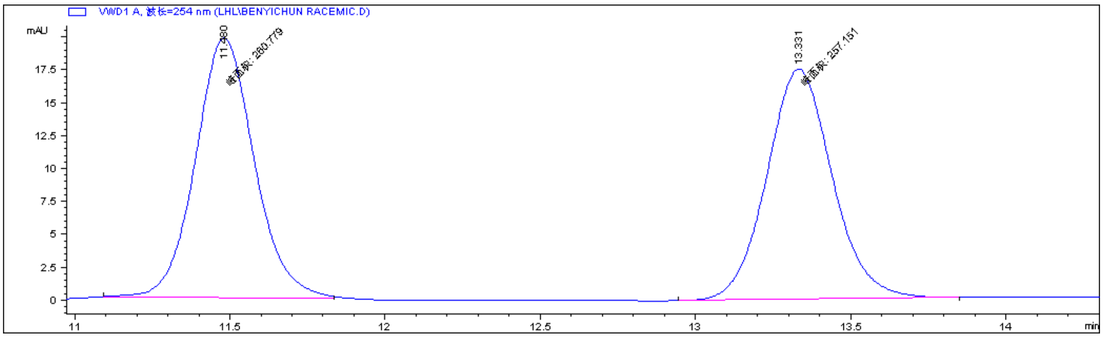

Figure S17. 1-Phenylethanol, colourless oil, racemic. HPLC conditions: 90.0:10.0 hexane/*i*-PrOH; flow rate 0.5 mL/min; t_r(major) = 11.4 min, t_r(minor) = 13.3 min.

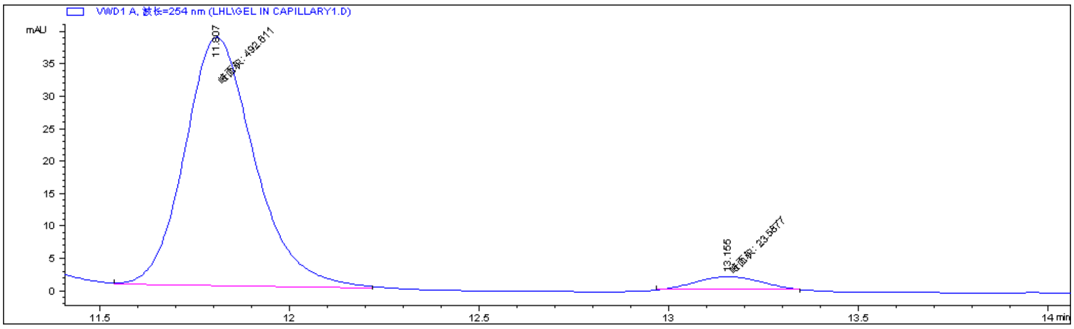

1st, 91% ee.

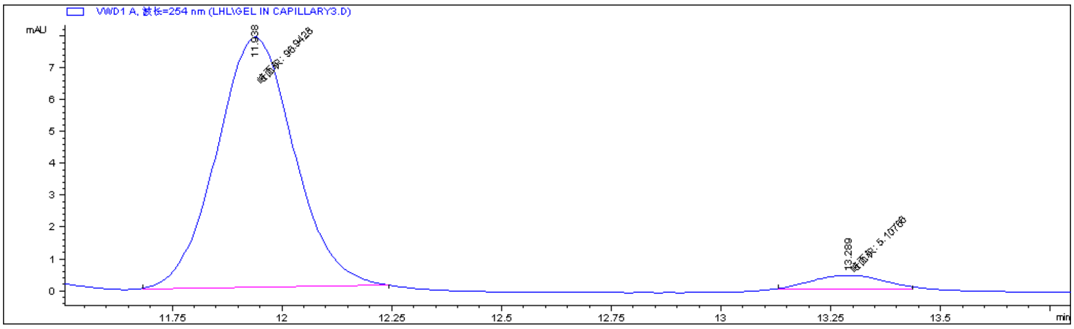

2nd, 90% ee.

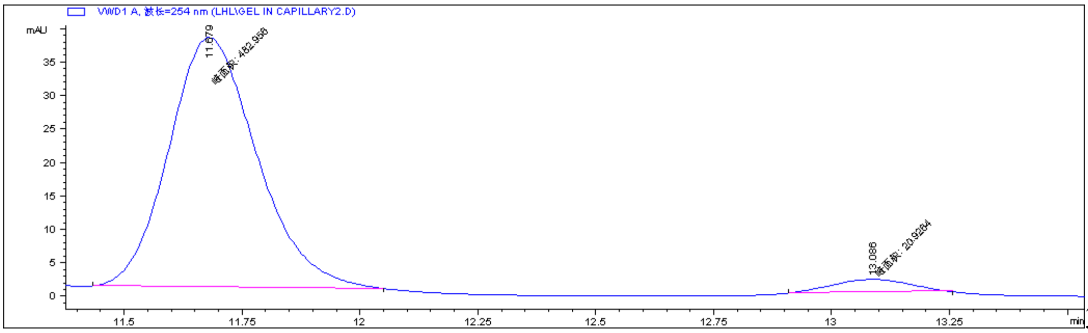

3rd, 92% ee.

Figure S18. 1-Phenylethanol obtained in the capillary reactor catalysed by Mn-salen imine gel (8 runs). (continued on next page)

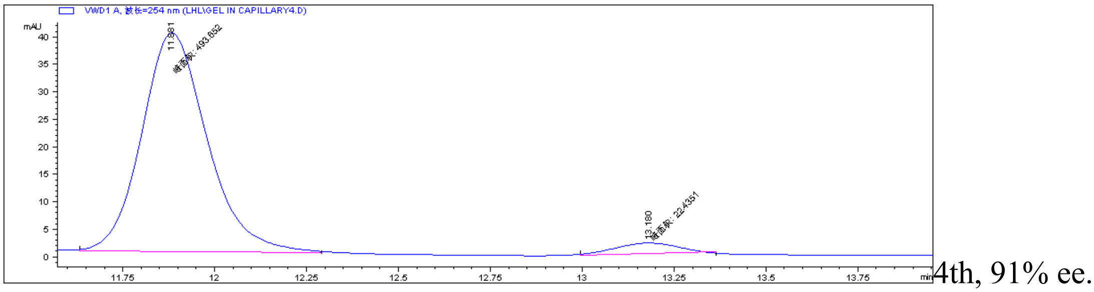

4th, 91% ee.

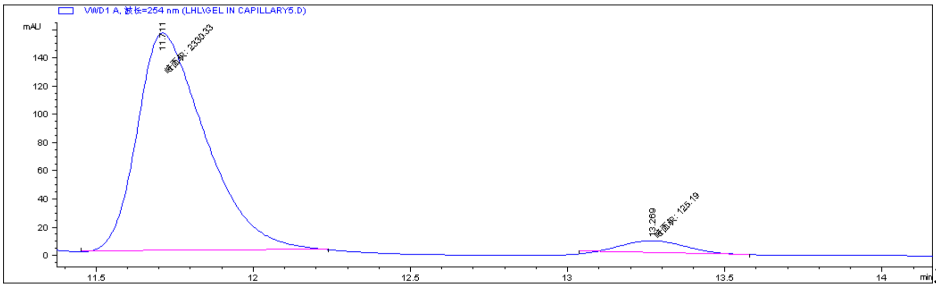

5th, 90% ee.

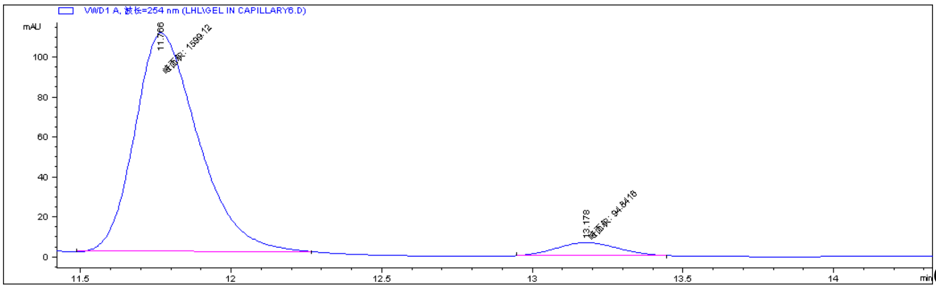

6th, 89% ee.

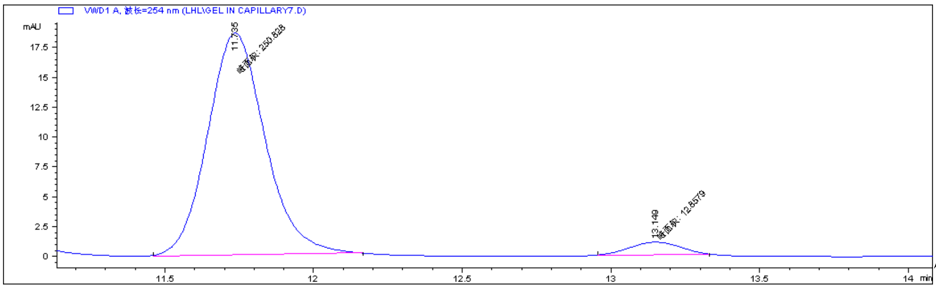

7th, 90% ee

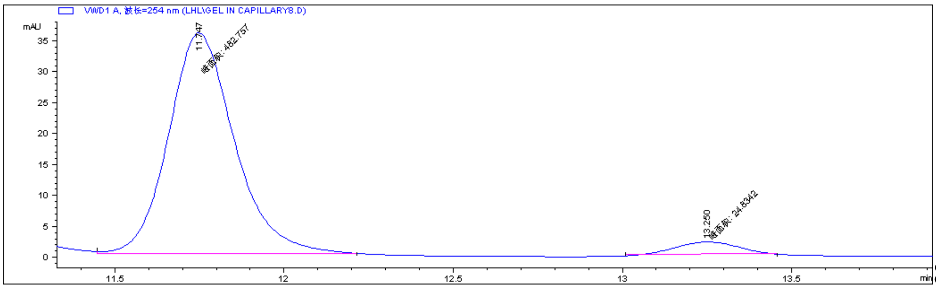

8th, 90% ee.

Figure S18. 1-Phenylethanol obtained in the capillary reactor catalysed by Mn-salen imine gel (8 runs).

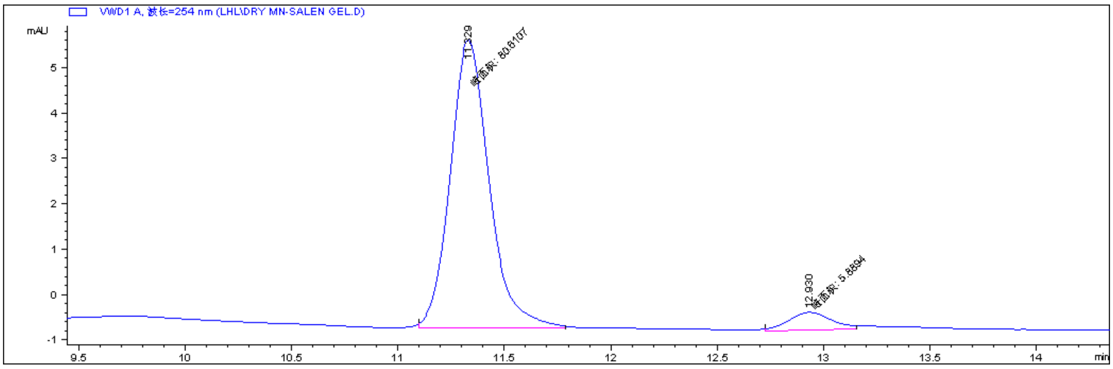

Figure S19. 1-Phenylethanol obtained in capillary reactor catalysed by Mn-salen imine xerogel.

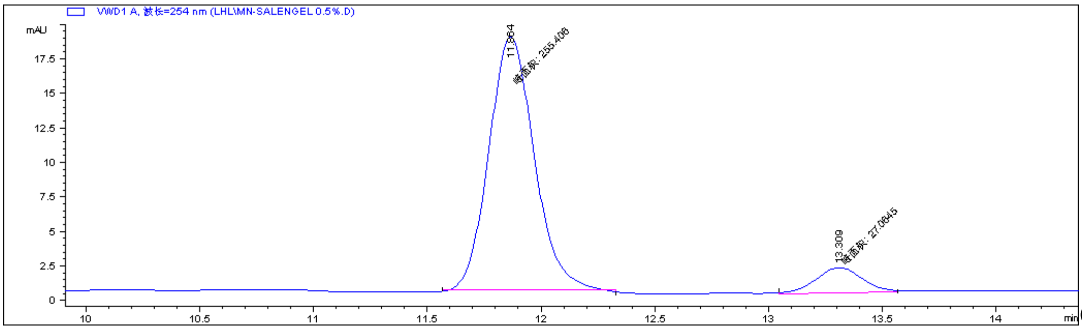

Cat. 0.5%, 80% ee.

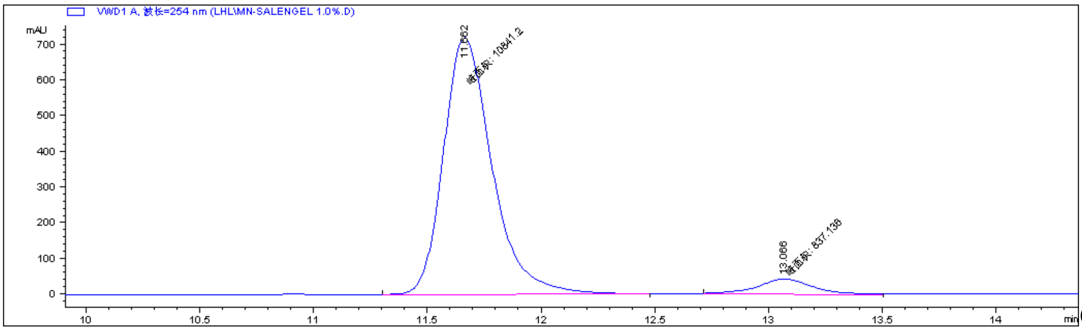

Cat. 1.0%, 86% ee.

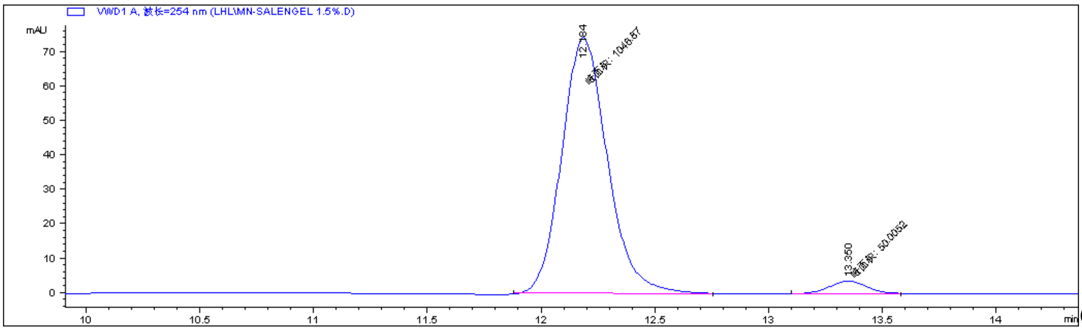

Cat. 1.5%, 91% ee.

Figure S20. 1-Phenylethanol obtained under batch conditions with various catalyst amounts catalysed by Mn-salen imine gel. (continued on next page)

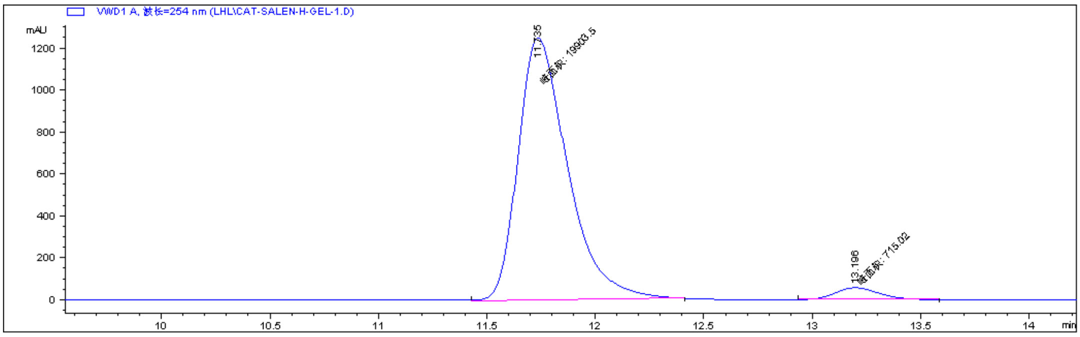

C

at. 2.0%, 93% ee.

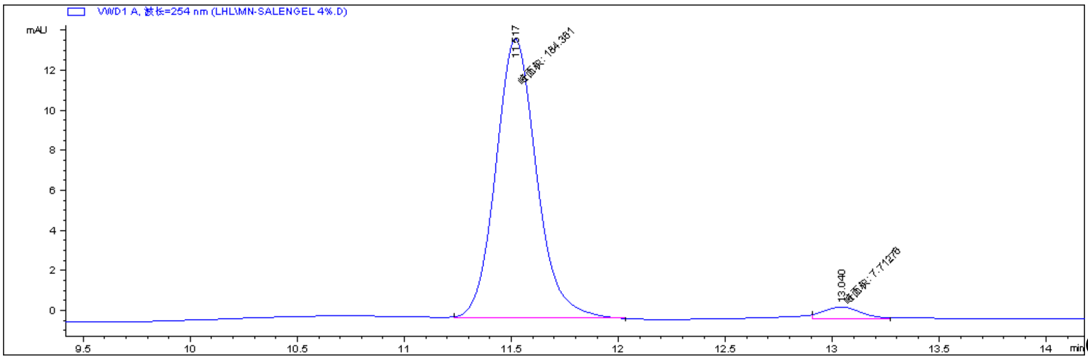

C

at. 4%, 92% ee.

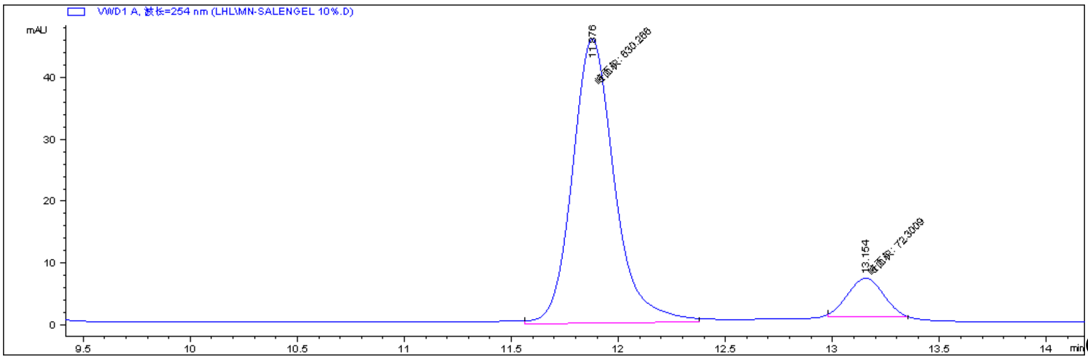

C

at. 10%, 77% ee.

Figure S20. 1-Phenylethanol obtained under batch conditions with various catalyst amounts catalysed by Mn-salen imine gel.

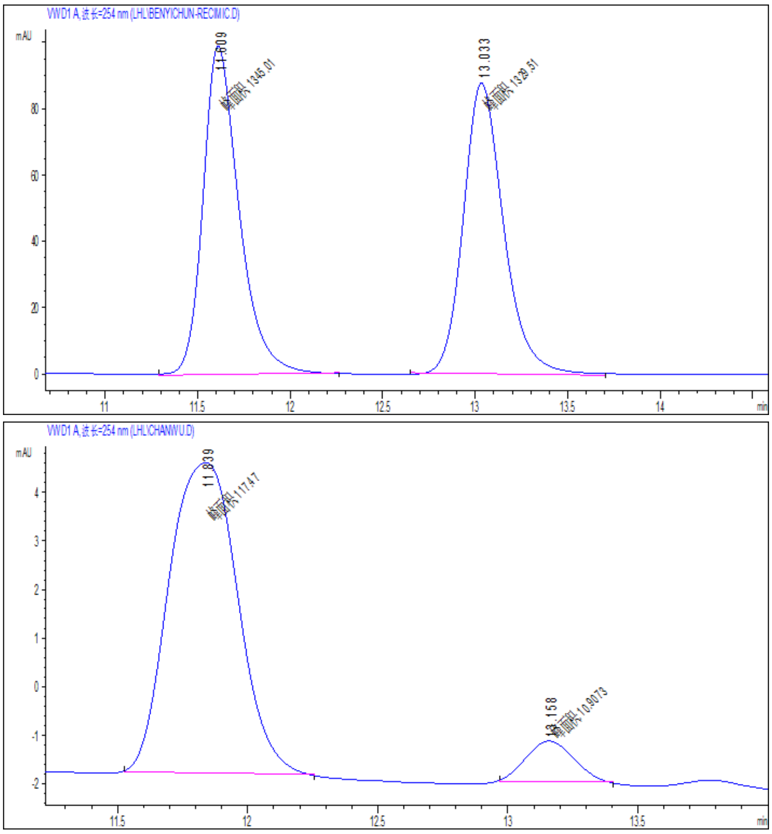

Figure S21. 1-Phenylethanol obtained under homogeneous conditions catalysed by B2-Mn. 83% ee. HPLC condition: 90/10 hexane/*i*-PrOH; flow rate 0.50 mL/min. t_r(major) = 11.60 min, t_r(minor) = 13.03 min.

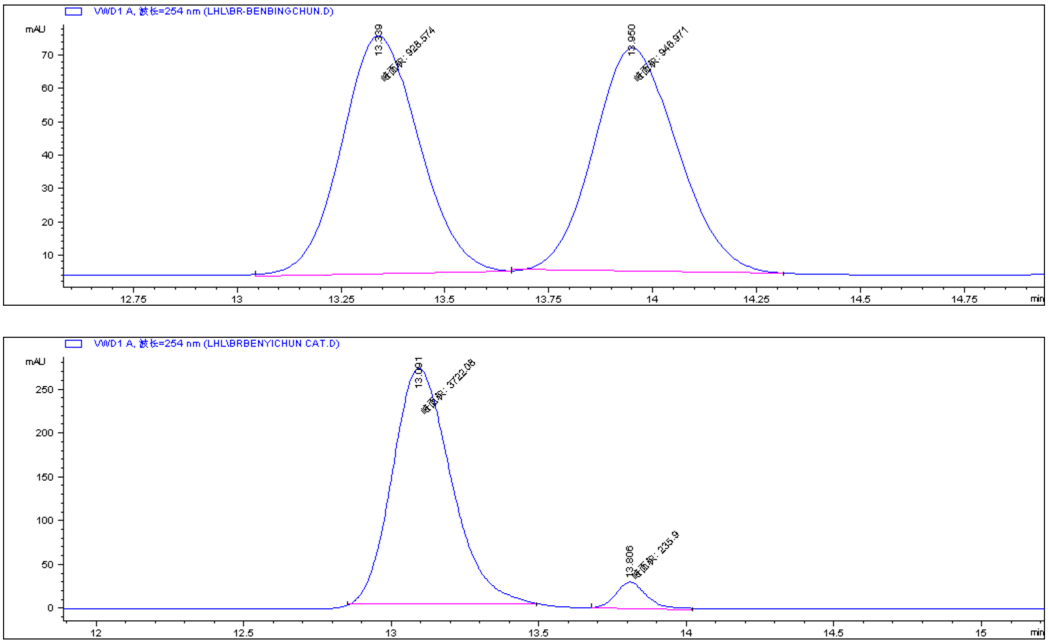

Figure S22. 1-(4-Bromophenyl)ethanol obtained in the capillary reactor catalysed by Mn-salen imine gel: colourless oil, 88% ee. HPLC conditions: 97.0:3.0 hexane/*i*-PrOH; flow rate 0.6 mL/min; t_r(major) = 13.3 min, t_r(minor) = 13.9 min.

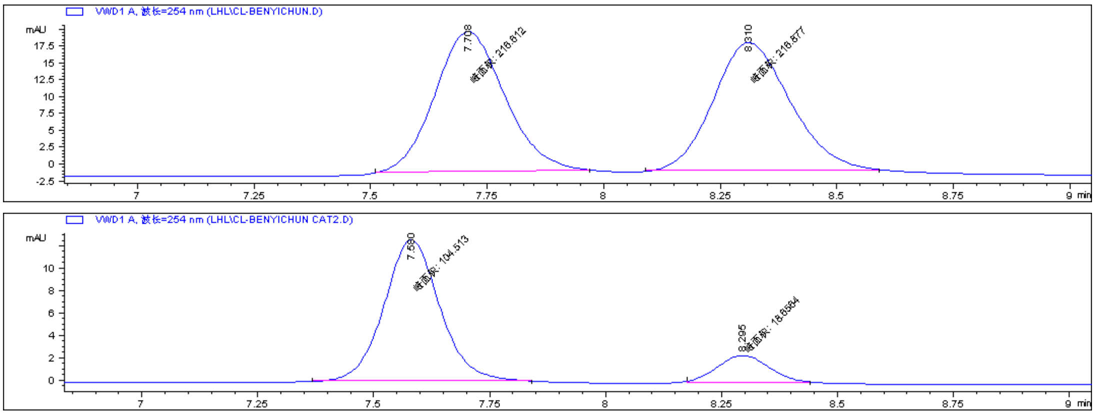

Figure S23. 1-(4-Chlorophenyl)ethanol obtained in the capillary reactor catalysed by Mn-salen imine gel: colourless oil, 70% ee. HPLC conditions: 95:05 hexane/*i*-PrOH; flow rate 1 mL/min; t_r(major) = 7.70 min, t_r(minor) = 8.3 min.

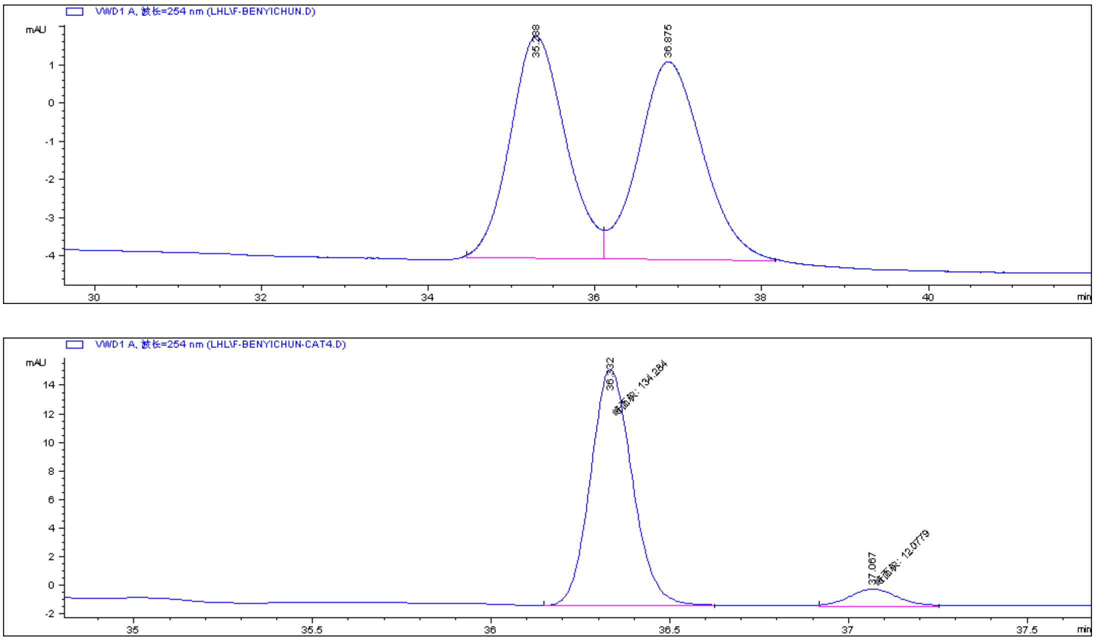

Figure S24. 1-(4-Fluorophenyl)ethanol obtained in the capillary reactor catalysed by Mn-salen imine gel: colourless oil, 82% ee. HPLC conditions: 99.5:0.5 hexane/*i*-PrOH; flow rate 1 mL/min; t_r(major) = 35.2 min, t_r(minor) = 36.8 min.

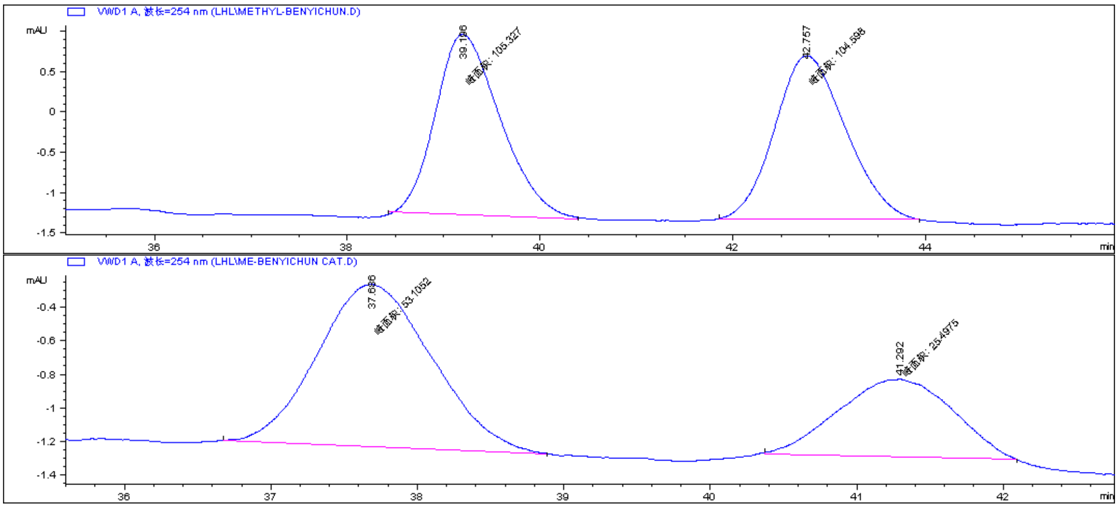

Figure S25. 1-(4-Methylphenyl)ethanol obtained in the capillary reactor catalysed by Mn-salen imine gel: colourless oil, 35% ee. HPLC conditions: 99.5:0.5 hexane/*i*-PrOH; flow rate 1 mL/min; t_r(major) = 39.2 min, t_r(minor) = 42.8 min.

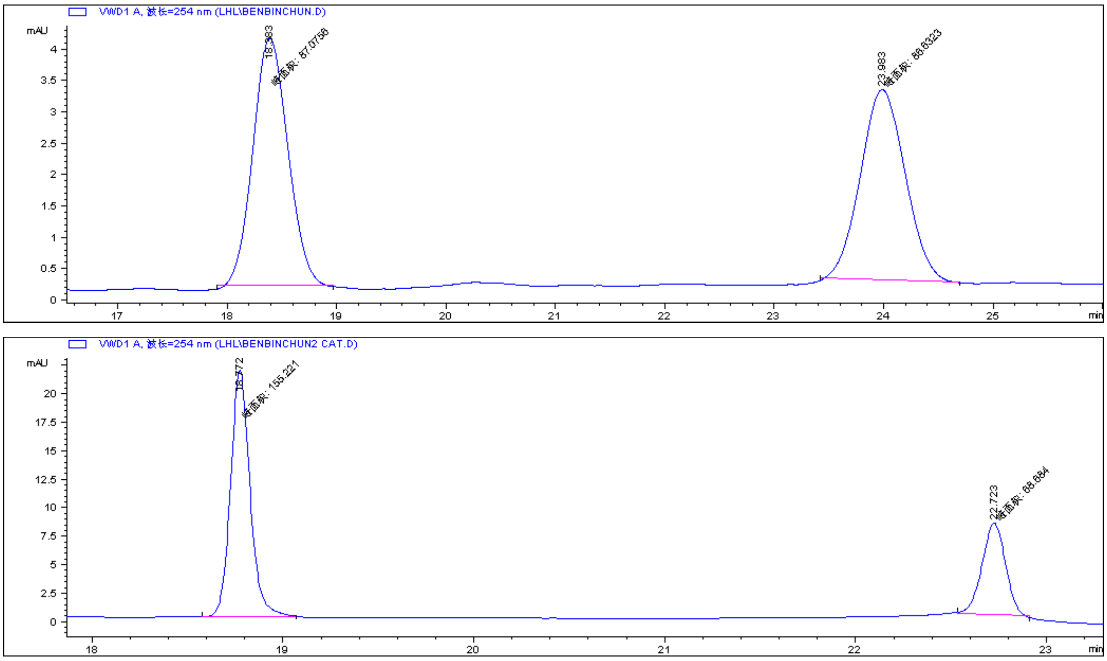

Figure S26. 1-Phenyl-1-propanol obtained in the capillary reactor catalysed by Mn-salen imine gel: colourless oil, 38% ee. HPLC conditions: 98.7:1.3 hexane/*i*-PrOH; flow rate 1.0 mL/min; t_r(major) = 18.4 min, t_r(minor) = 23.9 min.

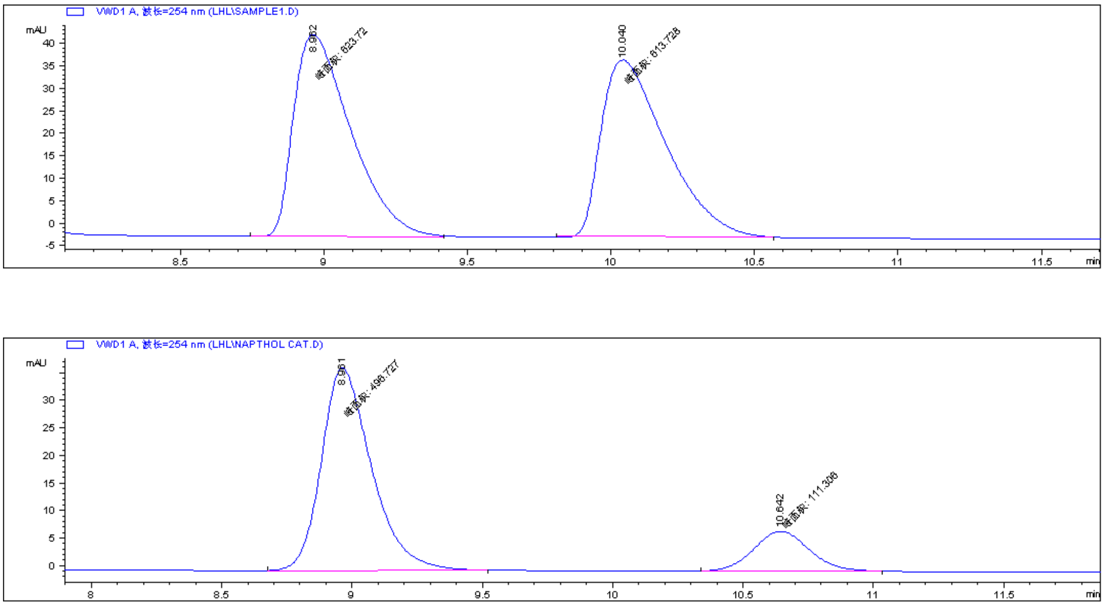

Figure S27. 1-Indanol obtained in the capillary reactor catalysed by Mn-salen imine gel: colourless oil, 62% ee. HPLC conditions: 90:10 hexane/*i*-PrOH; flow rate 0.8 mL/min; t_r(major) = 8.9 min, t_r(minor) = 10.0 min.

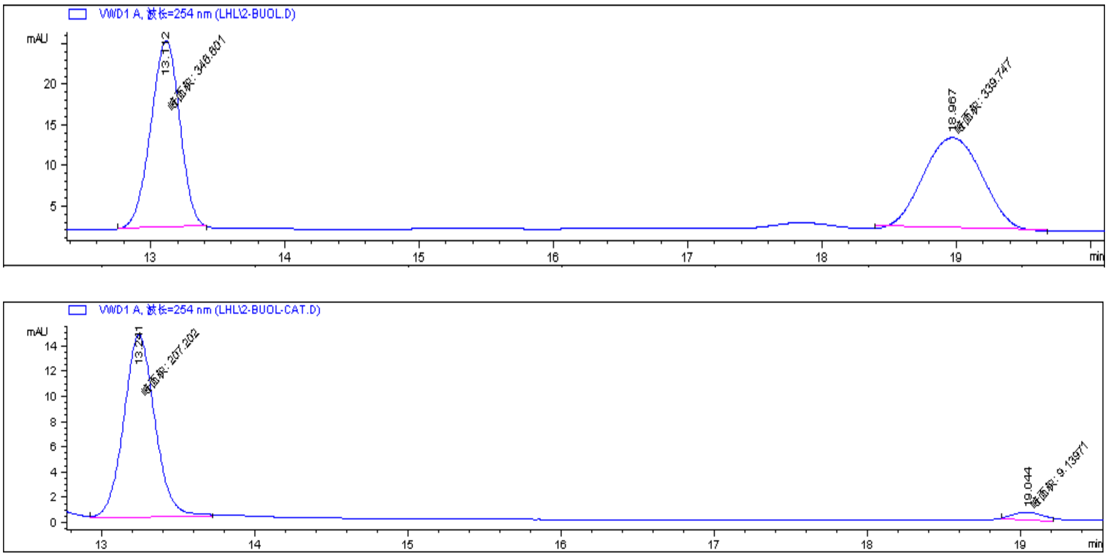

Figure S28. 4-Phenyl-2-butanol obtained in the capillary reactor catalysed by Mn-salen imine gel: colourless oil, 91% ee. HPLC conditions: 94.0:0.6 hexane/*i*-PrOH; flow rate 0.7 mL/min; t_r(major) = 13.1 min, t_r(minor) = 19.0 min.
